# Tenofovir-tethered gold nanoparticles as a novel multifunctional long-acting anti-HIV therapy to overcome deficient drug delivery-: an in vivo proof of concept

**DOI:** 10.1186/s12951-022-01750-w

**Published:** 2023-01-19

**Authors:** Leila Fotooh Abadi, Pramod Kumar, Kishore Paknikar, Virendra Gajbhiye, Smita Kulkarni

**Affiliations:** 1 Division of Virology, Indian Council of Medical Research-National AIDS Research Institute, Pune, 411 026 India; 2 Nanobioscience Group, Agharkar Research Institute, Pune, 411 004 India; 3 Department of Chemistry, Indian Institute of Technology, Mumbai, 400 076 India

**Keywords:** NanoART, Long-acting antiretroviral, Genotoxicology, HIV reservoirs, Preclinical, Biodistribution, In vivo imaging, Nanomedicine

## Abstract

**Background:**

The adoption of Antiretroviral Therapy (ART) substantially extends the life expectancy and quality of HIV-infected patients. Yet, eliminating the latent reservoirs of HIV to achieve a cure remains an unmet need. The advent of nanomedicine has revolutionized the treatment of HIV/AIDS. The present study explores a unique combination of Tenofovir (TNF) with gold nanoparticles (AuNPs) as a potential therapeutic approach to overcome several limitations of the current ART.

**Results:**

TNF-tethered AuNPs were successfully synthesized. Cell viability, genotoxicity, haemolysis, and histopathological studies confirmed the complete safety of the preparation. Most importantly, its anti-HIV1 reverse transcriptase activity was ~ 15 folds higher than the native TNF. In addition, it exhibited potent anti-HIV1 protease activity, a much sought-after target in anti-HIV1 therapeutics. Finally, the in vivo biodistribution studies validated that the AuNPs could reach many tissues/organs, serving as a secure nest for HIV and overcoming the problem of deficient drug delivery to HIV reservoirs.

**Conclusions:**

We show that the combination of TNF and AuNPs exhibits multifunctional activity, *viz.* anti-HIV1 and anti-HIV1 protease. These findings are being reported for the first time and highlight the prospects of developing AuNP-TNF as a novel next-generation platform to treat HIV/AIDS.

**Graphical Abstract:**

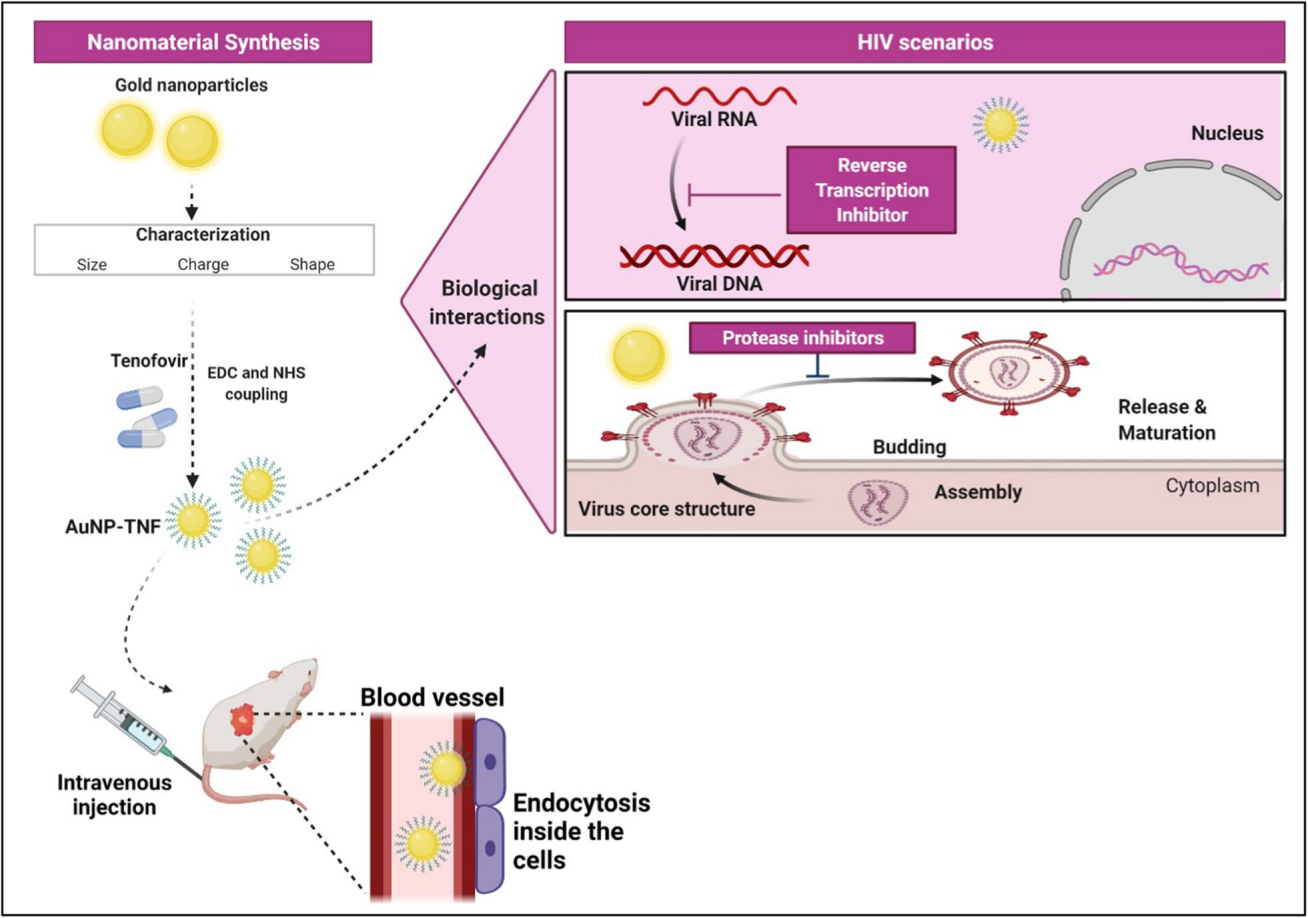

**Supplementary Information:**

The online version contains supplementary material available at 10.1186/s12951-022-01750-w.

## Background

The cure for Human Immunodeficiency Virus (HIV) indeed became a real possibility after the precedence set by three patients, *viz.* Timothy Ray Brown (the Berlin patient), Adam Castillejo (the London patient), and recently, a woman in New York. The focus, after that, shifted to intense investigation on eradicating the virus. Several human body parts are HIV niches, i.e., HIV reservoirs. Researchers soon realized that eliminating reservoirs of HIV would be a stumbling block to attaining the goal of virus eradication, especially on a massive scale.

It is well established that incorporating Antiretroviral Therapy (ART) into clinical platforms increases life expectancy and quality. Furthermore, combination Antiretroviral Therapy (cART) targets distinct phases of the HIV replication cycle and improves overall antiviral efficacy to > 80%, even in drug-resistant cases [1–3]. However, a complete remedy for HIV remains elusive.

Given these limitations, many patients require lifelong ARTs, which entails long-term drug toxicity. Also, there is a lot of patient reluctance to treatment adherence, which increases the risk of developing drug resistance. In addition, ARTs notoriously fail to penetrate specific tissues, allowing viral reservoirs to form. Therefore, despite all the advantages of ARTs, further substantial improvement in their usage is the need of the hour.

HIV reservoirs in infected individuals receiving ARTs are attributable to several mechanisms, including intrinsic stability of latently infected resting CD4^+^ T cells, the periodic homeostatic proliferation of cells that sustain the latent viral reservoir, and low penetration of ARTs into the tissue where the virus replicates [4, 5]. Recent comprehensive molecular investigations on the prevalence of viral integration sites in the genomes of infected patients receiving ART offer key insights into the potential mechanisms responsible for the occurrence of HIV reservoirs. These findings imply that the location of viral integration in the human genome influences the establishment and sustainability of HIV reservoirs. Despite these critical insights on the molecular mechanisms of reservoir formation, inadequate penetration of ARTs into the reservoir sites and their consequent failure to reduce the viral load remain an unmet challenge in the conquest against HIV [6–8]. Over the past decade, harnessing the potential of the cutting-edge science of nanotechnology in anti-HIV therapy has become an attractive proposition [9]. Drug Delivery Systems (DDS) utilizing nanomaterials are being developed as high payload, single-dose systems to boost the pharmacodynamics and pharmacokinetics of ARTs (nano-ART). Surface modification ensuring slow-release of the drug payload and its receptor-mediated targeting is the most sought-after feature of the nano-ARTs [10–12]. Among various types of nanomaterials, metal-based nanoparticles have been thoroughly investigated for their potential antimicrobial activity [13–18].

Silver, gold, copper, iron, titanium dioxide, zinc oxide, etc., are the main metal nano agents, which are considered mainly for studies thus far and they have yielded promising findings. However, gold nanoparticles (AuNPs) have attracted much interest due to their broad effectiveness against pathogenic microorganisms. AuNPs are a promising plasmonic material with size- and shape-dependent optical, electronic, and physicochemical properties. AuNPs are already used in gene therapy, imaging, targeting, and delivery of therapeutics owing to these intrinsic properties [19–25]. In fact, their use in targeted cancer therapy has reached the clinical trial stage [26]. However, reports on AuNPs as carriers for anti-HIV1 therapeutics are scarce [27–30]. Limited exciting studies proposed that AuNPs inhibit HIV replication via fusion/viral entry or integrase [31, 32]. Attaching twelve molecules of TAK-779, a C-C chemokine receptor type 5 (CCR5) antagonist, to one gold nanoparticle restored the drug's efficacy in combating HIV infection in vitro [31]. This result suggests a potentially promising approach for repositioning the available drugs with low bioavailability to the clinical platform de novo.

Tenofovir [9-[-(R)-2(phosphonomethoxy) propyl] adenine] (TNF), a precursor of Tenofovir Disoproxil Fumarate (TDF), is marketed commercially under VIREAD^®^. Tenofovir alafenamide (TAF) is also proposed as another prodrug that has improved properties compared to TDF. It was observed that TAF-containing regimens show a 90% decline in TNF plasma concentrations and enhanced renal and bone safety. In contrast, TDF-containing regimens are linked with changes in bone mineral density decline, renal function, and the rare occurrence of severe adverse renal events, including Fanconi syndrome [33]. TNF is a nucleotide analogue reverse transcriptase inhibitor that works against the CCR5 and CXCR4 tropic HIV-1 strains and can be safely taken orally and vaginally using Intra-Vaginal Rings (IVRs) and gels [34–40]. The latter could help alleviate some of the adverse effects of the daily oral dose. Studies show that women who applied 1% TNF gel intravaginally before and after sexual intercourse had a 39 percent lower risk of contracting HIV [41, 42]. Furthermore, TNF is prescribed with other antiretroviral drugs to treat adult patients infected with HIV [43]. The administration of the recommended oral dose (300 mg) of TNF might be one of the reasons for displaying the associated adverse effects in HIV/Acquired Immunodeficiency Syndrome (AIDS) patients due to TNF's extended biological half-life [44]. Also, it is available in combination with Emtricitabine/efavirenz (Atripla), Emtricitabine (Truvada), and Emtricitabine/rilpivirine (Complera) [45]. TNF is hydrophilic; however, it demonstrates low oral bioavailability (i.e., 25–30%) in vivo [46].

It is evident from the chemical structure of TNF that it exhibits a negative charge facilitating improved systemic transport and disposition to various tissues/organs [47–50]. Therefore, drug delivery strategies for TNF are the key to the low bioavailability and toxicity concerns [51]. TNF encapsulation inside modified long-circulating liposomes has recently been described to deliver this hydrophilic anti-HIV1 medicine to the reticuloendothelial system for improved treatment effectiveness [52]. Against the backdrop of the information given above, it was thought worthwhile to develop AuNPs as nanocarriers for TNF. Herein, we report successful synthesis and characterization of TNF conjugated AuNPs using EDC/NHS coupling method with improved bioavailability and potent anti-HIV1 activity.

## Results

### Characterization

Adequate structural and chemical characterization of the AuNPs is essential while considering their utilization as a drug cargo. Hence, the free TNF, AuNPs, and TNF conjugated AuNPs (AuNP-TNF) were characterized for their size, morphology, structure, and distribution of their elements.

**Fig. 1:**
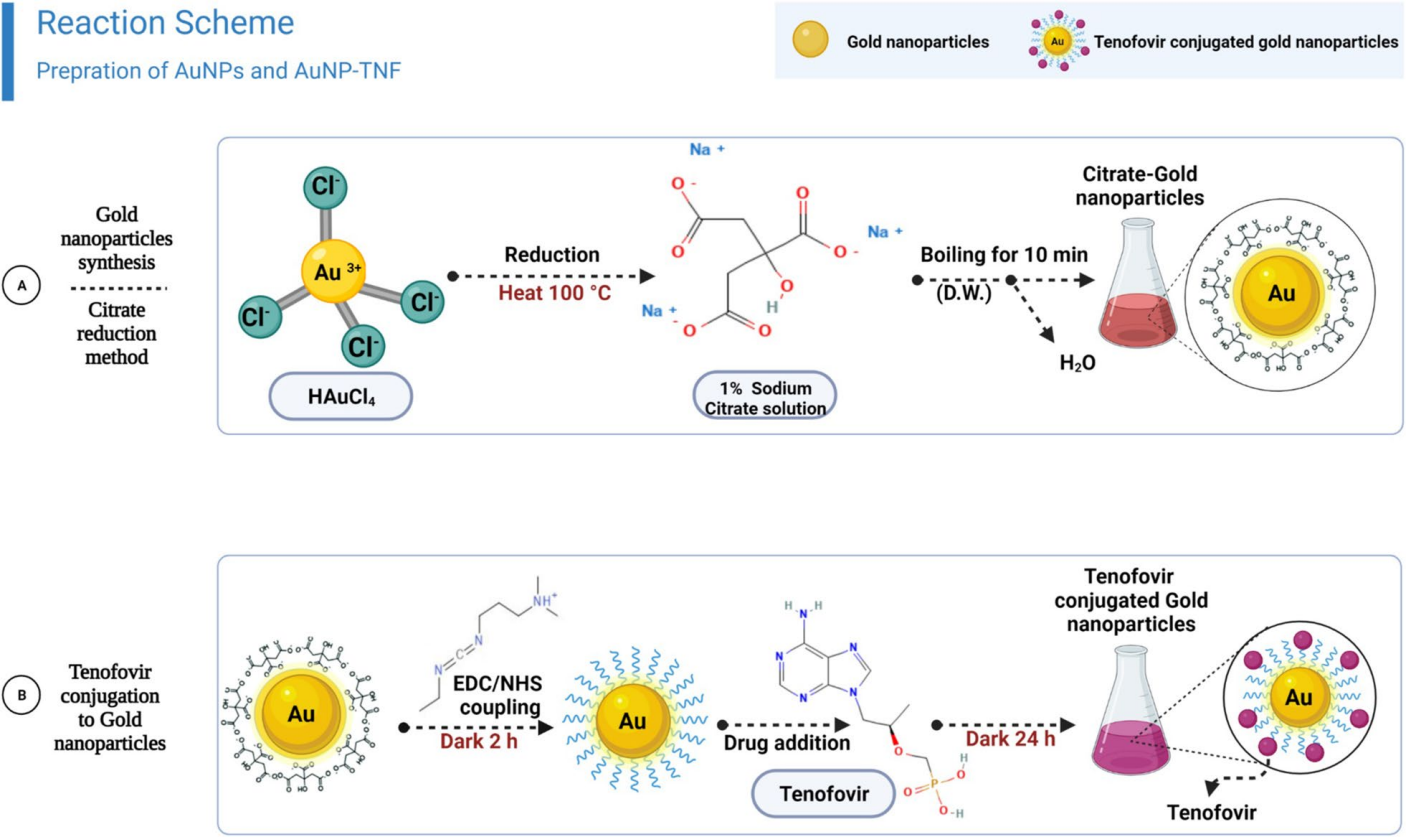
A schematic diagram of; **A** gold nanoparticles synthesis using citrate reduction method and **B** reaction scheme shows the covalently binding of EDC/NHS on the surface of the gold nanoparticles, followed by conjugation to free amine of the target molecule, Tenofovir

The hydrodynamic size of AuNPs was determined to be 26.53 nm (Polydispersity Index (PDI)- 0.144) with a net negative surface charge of -41.1 ± 5.94 mV which could be attributed to the carboxyl groups of the citrate ions capping the AuNPs (Fig. 1A and B). The D10, D50, and D90 values were 15.1, 21.8, and 34.0 nm for AuNPs. The standard deviation for AuNP size analysis was found to be 3.26 nm.

The measured hydrodynamic size for AuNP-TNF was 37.59 nm (PDI- 0.172) with a negative charge of - 47.0 ± 5.77 mV (Fig. 1C, and D). The standard deviation for AuNP-TNF size analysis was found to be 4.46 nm. After that, the measured hydrodynamic size for Cy5.5-AuNP was 31.78 nm (PDI- 0.160) with a negative charge of -19.6 ± 4.56 mV (Additional file 1: Fig. S7 A, and B). The standard deviation for AuNP-TNF size analysis was found to be 4.8 nm.

### Field emission-scanning electron microscopy (FE-SEM) analysis

**Fig. 2:**
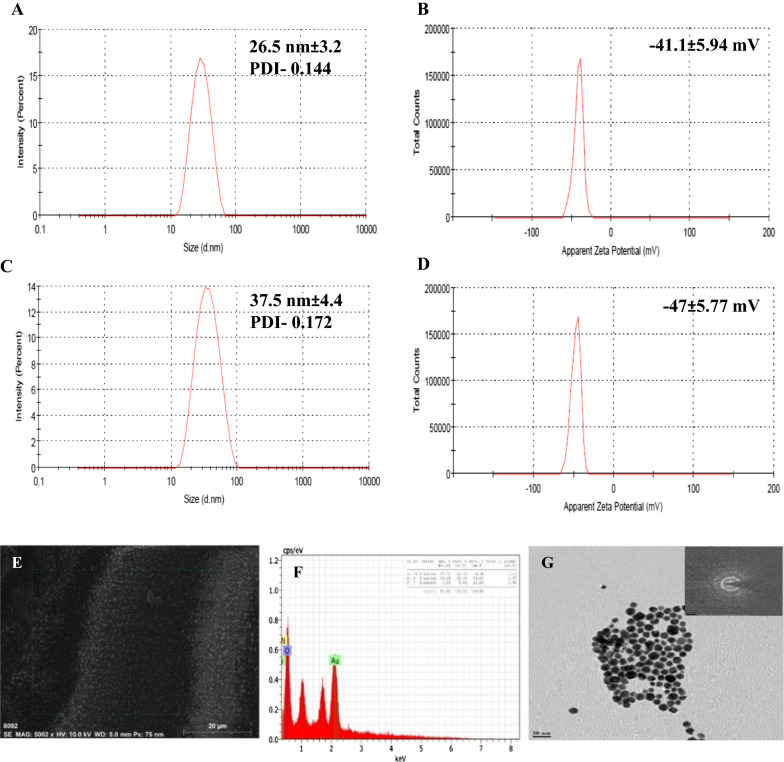
**A** Size and **B** Surface charge as measured by DLS of AuNPs, and **C** Size (Dynamic light scattering) and **D** Surface charge (Zeta) of AuNP-TNF, **E** Field Emission-Scanning Electron Microscopy, **F** EDS, and **G** Transmission Electron Microscopy [SAED image in inset] of AuNPs

The FE-SEM analysis was used to determine the morphology of the synthesized AuNPs. As shown in Fig. 2E, the AuNPs were nearly spherical and ≈ 25 nm in size. The Energy Dispersive Spectroscopy (EDS) spectra (D) and elemental mapping of Au nanoparticles and overlap elements are shown in Additional file 1: Fig. S2. These figures indicated the existence of Au, and O in the synthesized nanoparticles. Based on these findings, a semiquantitative approach evaluated the Au content of the produced powder to be around 53.70 wt %, confirming that the synthesis process was adequate. AuNPs had the highest peak in the Energy Dispersive X-Ray Analysis (EDX) spectra of nanoparticles at 1.12 keV. The other overlapping ions were evident in the EDX spectra, which are tied to the lattice and the dispersion medium.

### Transmission electron microscopy (TEM) and Selected area electron diffraction (SAED) analysis

According to TEM observation, the synthesized AuNPs and AuNP-TNF were spherical with a average size of 24.30 ± 2.73 nm (Fig. 2G) and 30.75 ± 2.92 nm (Additional file 1: Fig. S8), respectively. Using SAED, the crystallinity of AuNPs was examined. Figure 2G (Insert) depicts the SAED pattern of the AuNPs. According to the face-centered cubic structure of synthesized gold nanoparticles, the spots in the SAED pattern were indexed and revealed that the particles were single and crystalline. The other overlapped ions that appeared in the EDX spectrum were found to be associated with the TEM grid and the dispersion medium, respectively (Fig. 2F).

### Drug conjugation

Further, the conjugation of TNF to AuNPs resulted in approximately 16% (weight %), TNF being tethered to the AuNPs surface as determined from the *UV-Vis* spectrophotometer analysis. The Thermogravimetric Analysis (TGA) results (Additional file 1: Fig. S4) supported the effective conjugation of TNF to the AuNPs. The initial weight loss, i.e., below 150 ℃, is attributed to the adsorbed water molecules on the nanoparticles. Thus, the weight loss (~ 11%) in AuNP-TNF observed at 150-800 ℃ is due to the loss of TNF molecules tethered to the AuNPs' surface.

### UV-Visible (*UV–-Vis*) spectroscopy

*UV-Vis* spectroscopy was used to examine the optical characteristics of the AuNPs-based nanoconjugates that had been synthesized. The visible spectrum analysis was from 400-700 nm wavelength. The AuNPs solution with a wine-red tint indicated the maximum Surface Plasmon Resonance (SPR) peak at 524 nm (Additional file 1: Fig. S1, turquoise peak). In comparison, the maximum absorption peak was found to be shifted to 526 nm (Additional file 1: Fig. S1, green peak) in the case of AuNP-TNF, which suggested an increase in size due to TNF conjugation.

### Fourier Transform-Infrared (FT-IR) spectroscopy analysis

The FT-IR spectra, shown in Additional file 1: Fig. S3, displayed a broad peak of a carboxyl group from 2800–-300 cm^−1^ for AuNPs. Whereas spectrum AuNP-TNF showed a narrower peak centered around 3280 cm^−1^, which might be traced back to the -NH stretch of secondary amide, suggesting the formation of a secondary amide bond between AuNPs and TNF. Further, the spectrum of AuNP-TNF displayed a peak for -NH bending at 1570 cm^−1^ and a peak for C-N stretch at 1296 cm^−1^, confirming the successful formation of an amide bond between AuNPs and TNF.

### In vitro* investigations*

#### Cellular viability

**Fig. 3:**
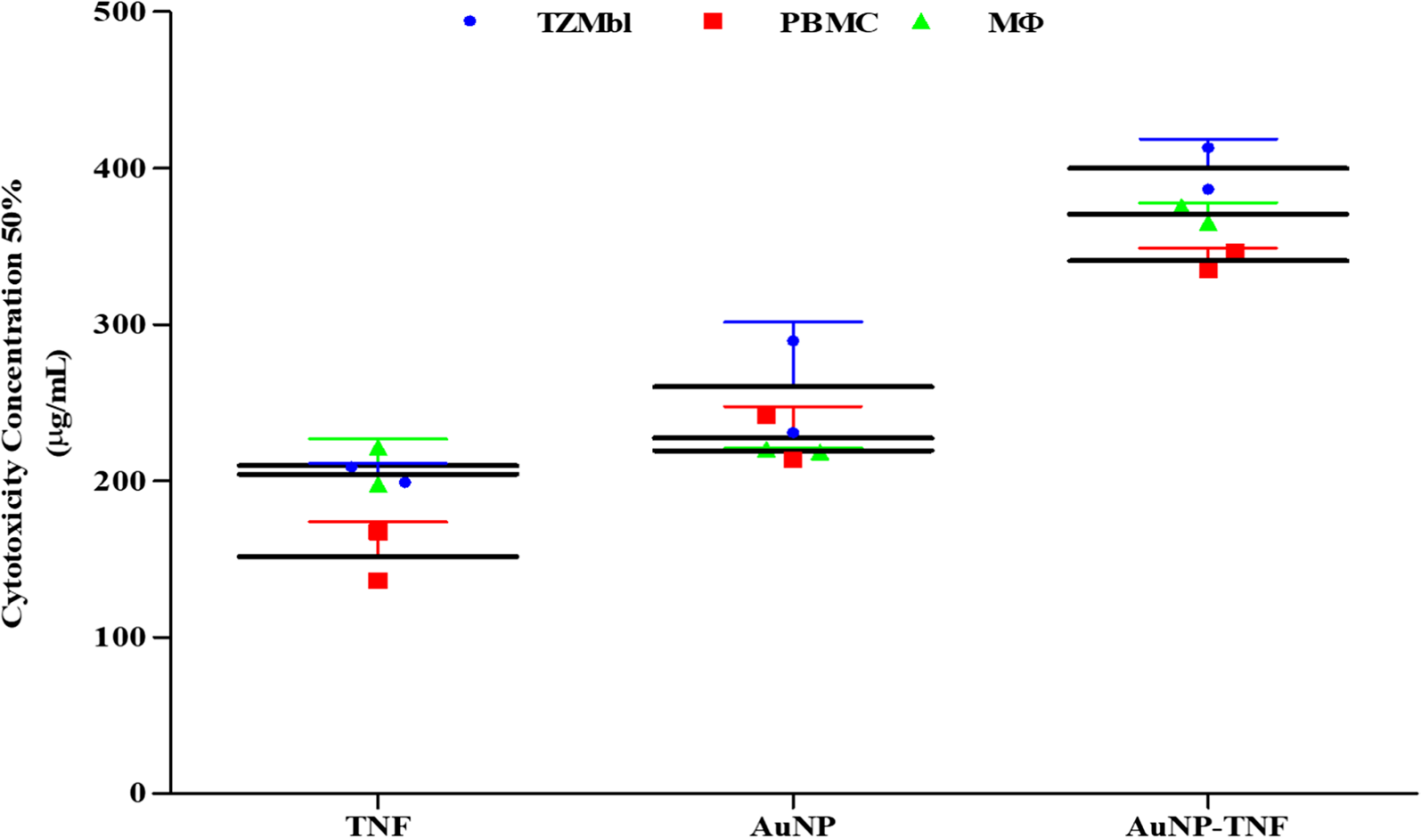
In vitro cell viability assessment, the graph preseing the fifty percent cytotoxicity concentration (CC_50_, µg/mL) of free TNF, AuNPs, and AuNP-TNF in TZM-bl, PBMCs, and MΦ cells

The 2,5-diphenyl-2H-tetrazolium bromide (MTT) cell proliferation assay was used to evaluate the cellular viability of free TNF, AuNPs, and TNF conjugated AuNPs, and the data were interpreted as CC_50_ (Fig. 3). Data presented show higher CC_50_ values for all the test materials in TZM-bl (48 h), Peripheral Blood Mononuclear Cells (PBMC), and MΦ cells (5 days). Remarkably, even after a prolonged incubation period, the viability of the AuNP-TNF-treated cells was significantly greater than those treated with free TNF (p ≤ 0.05). However, with regard to AuNPs, higher cell viability was observed as compared with the free drug (TNF) at the various tested concentrations (Additional file 1: Fig. S5).

#### Hemolysis

The results (Additional file 1: Fig. S6) revealed that the AuNPs could elicit hemolysis and severely impact Red Blood Cells (RBCs) viability at 20 μg/mL concentration compared with the free drug and bare nanoparticles. The cut-off point for assessing hemolysis according to the criterion in the ASTM E2524-08 standard, is 5% [53].

#### Genotoxicity

**Fig. 4:**
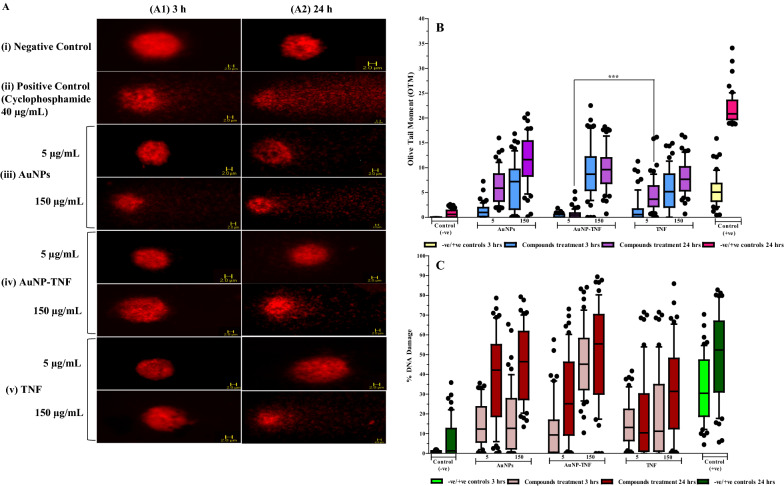
**A** Columns A1 and A2 represent fluorescent microscope images (40X) of the Ethidium bromide (EtBr)-stained cells treated with compounds and controls, **i **Represent Negative control, **ii** Represent positive control, cells treated with 40 µg/mL Cyclophosphamide, **iii** Represent cells treated with AuNPs, **iv** Represent cells treated with AuNP-TNF, and **v** Represent cells treated with TNF, **B/C **Box & Whiskers plots of comet assay in PBMCs. **B** Olive Tail Moment (OTM), **C** %DNA Damage. The solid line indicates the median for each group. The box edges mark the 25th and 75th percentiles of the observed values, and the T-bars indicate the 10th and 90th percentiles. (*** denotes p≤0.0001, the significant difference is in comparison with the drug control, TNF)

Shows Fig. 4A the fluorescent microscope images (40X) of the Ethidium bromide (EtBr)-stained cells treated with free TNF, AuNPs, and AuNP-TNF at the concentration of 5 and 150 µg/mL at two-time points (3 and 24 h). The cells treated with 40 µg/mL Cyclophosphamide, an anti-neoplastic chemotherapy drug that can induce Deoxyribo-Nucleic Acid (DNA) damage, served as a positive control (+ ve). The untreated cells served as a negative control (-ve). The fluorescence microscopy analysis of a single cell at 3 h post-treatment (Fig. 4A1) at both concentrations revealed no significant increase in tail length compared to the + ve control.

In contrast, 24 h post-treatment (Fig. 4A2) showed a substantial increase in a single tail length and DNA strand breaks when the cells were treated with 150 µg/mL of compounds. However, the extent of DNA damage was lower than the positive control. Figure 4B and C show extensive and dose-dependent damage to DNA after treatment of the cells with different concentrations of the compounds.

#### Detection of apoptosis, using FCM

**Fig. 5:**
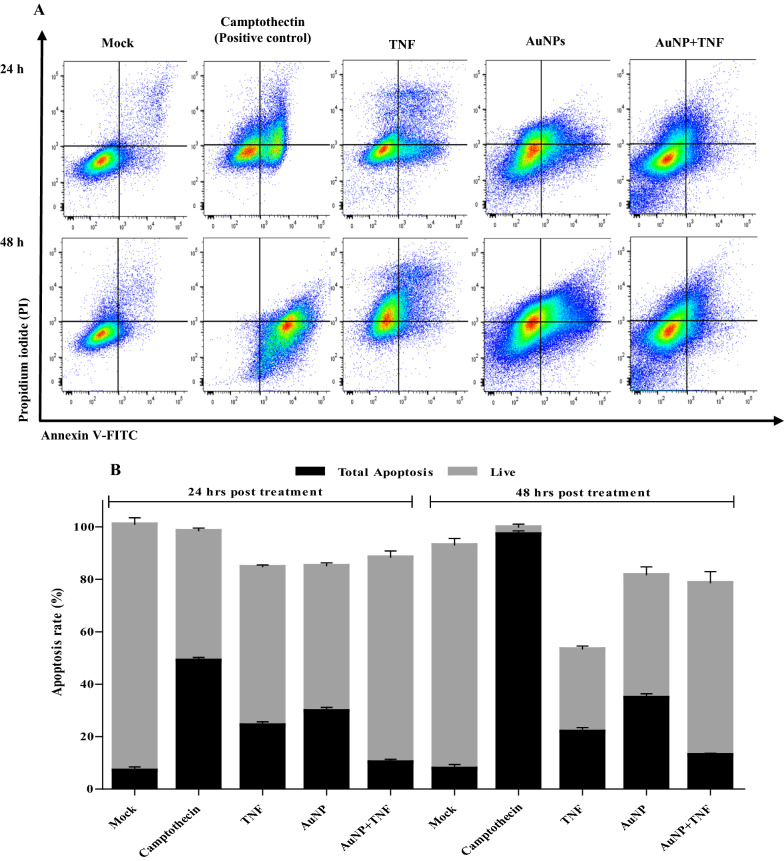
**A** Flow cytometry to determine the cell death for apoptosis. Effect of 80, 420, and 500 µg/mL of free TNF, AuNPs, and AuNP-TNF were analyzed at 24 h and 48 h, respectively, **B** The total apoptosis rate [early+late apoptosis] in the treated and untreated TZM-bl cell line

This experiment was conducted to strengthen the toxicity studies. The TZM-bl cells were treated with the free TNF, AuNPs, and AuNP-TNF (80, 420, and 500 µg/mL) incubated for 24 and 48 h. Figure 5A and B show the flow cytometry analysis of treated TZM-bl cells with respective controls at both time points. The nano-ART's total apoptosis rate/live population data compared to the other study groups showed a lower apoptosis rate at both time points. Interestingly, even after 48 h of exposure, the AuNP-TNF percent apoptosis rate was nearly half that of free TNF.

#### Assessment of anti-HIV1 activity

**Fig. 6:**
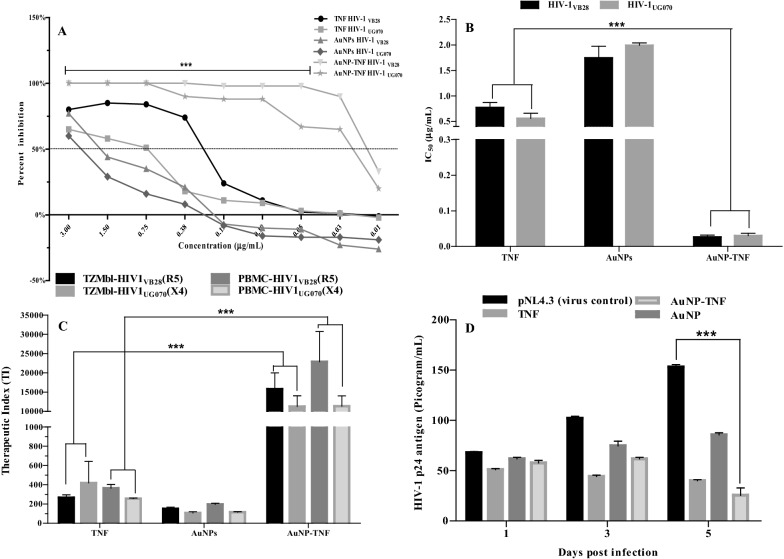
**A** Percent inhibition of HIV1_VB28_ and HIV1_UG070_ of free TNF, AuNPs, and AuNP-TNF in TZM-bl cell line with different concentrations, **B** Inhibitory concentrations (IC_50_) of infected PBMCs treated with compounds against HIV-1 strains were used in this study, **C** Therapeutic index (TI) of free TNF, AuNPs, and AuNP-TNF in TZM-bl cell line & PBMCs associated with both HIV-1 strains, **D** HIV-1 p24 Gag antigen level (pg/mL) in 1, 3, and 5 days post-infection in MΦ exposed to the compounds. (***denotes p ≤ 0.0001, the significant difference is in comparison with the drug control, TNF)

TZM-bl and PBMC cells infected with two distinct primary isolates (HIV1_VB28_ (R5) or HIV1_UG070_ (X4)) were exposed to various sub-toxic concentrations of free TNF, AuNPs, and AuNP-TNF to assess potential anti-HIV1 efficacy. Table 1 shows the half-maximal inhibitory concentration (IC_50_) values for free TNF, AuNPs, and AuNP-TNF against HIV1_VB28_ and HIV1_UG070_ strains in TZM-bl cells and PBMCs, respectively. It was confirmed that the TNF anti-HIV1 activity was significantly enhanced upon conjugation with AuNPs in TZM-bl cells and PBMCs. (Fig. 6A and B) Similarly, the HIV-1 p24 antigen level (pg/mL) in infected MΦ cells (Fig. 6D) revealed that the AuNP-TNF was able to inhibit the HIV-1 NL4-3 molecular clone (pNL4-3). The Therapeutic Indexes (TIs) for free TNF, AuNPs, and AuNP-TNF in TZM-bl and PBMCs cells infected with HIV-1 strains were subsequently computed. (Table 1 and Fig. 6C). Considering AuNP-TNF has a lower IC_50_ value and higher 50% cytotoxic concentration (CC_50_, drug/compound concentrations necessary to impair cell viability by 50%), it achieves a greater TI over free TNF (p ≤ 0.0001). These results confirmed that the synthesized nano-ART could inhibit both HIV-1 primary isolates as well as pseudovirus efficiently compared to free TNF (Fig. 6).

**Table 1:** IC_50_ and TI of free TNF, AuNPs, and AuNP-TNF in TZM-bl & PBMCs

Sr. No	Compound name	Anti–HIV1 assay TZM-bl cells				Anti–HIV1 assay PBMCs			
		IC_50_ HIV1_VB28_ (R5) (μg/mL)	TI HIV1_VB28_ (R5)	IC_50_ HIV1_UG070_ (X4) (μg/mL)	TI HIV1_UG070_ (X4)	IC_50_ HIV1_VB28_ (R5) (μg/mL)	TI HIV1_VB28_ (R5)	IC_50_ HIV1_UG070_ (X4) (μg/mL)	TI HIV1_UG070_ (X4)
1	TNF	0.771 ± 0.080	264.68	0.570 ± 0.196	358.29	0.394 ± 0.053	364.31 ± 30.48	0.560 ± 0.083	255.35 ± 5.19
2	AuNPs	1.745 ± 0.187	149.12	2.427 ± 0.079	107.20	1.171 ± 0.067	196.23 ± 8.32	0.1993 ± 0.039	115.01 ± 3.6
3	AuNP-TNF	0.026 ± 0.005	15,178.53 (***)	0.037 ± 0.008	10,796.86 (***)	0.016 ± 0.004	22,897.63 ± 6375.34 (***)	0.030 ± 0.005	11,332.61 ± 2198.59 (***)

*** denotes p≤0.0001, the significant difference is in comparison with the drug control, TNF

#### In vitro off-target activity evaluation to elucidate the mechanism of action

##### Anti-HIV1 Reverse Transcriptase (RT-ase) activity detection

In vitro inhibition of HIV-1 reverse transcriptase by free TNF, AuNPs, and AuNP-TNF was investigated. The IC_50_ values were derived using non-linear regression curves based on the inhibition of the virus at various concentrations of the compounds and (compared with controls without inhibitors). The IC_50_ values of free TNF, AuNPs, and AuNP-TNF were found to be 0.98 ± 0.072, 5.32 ± 0.860, and 0.067 ± 0.005 µg/mL, respectively, which demonstrated that the conjugation of TNF with nanoparticles has a substantial inhibitory effect on the RT-ase enzyme.

##### Anti-HIV1 protease activity detection

**Fig. 7:**
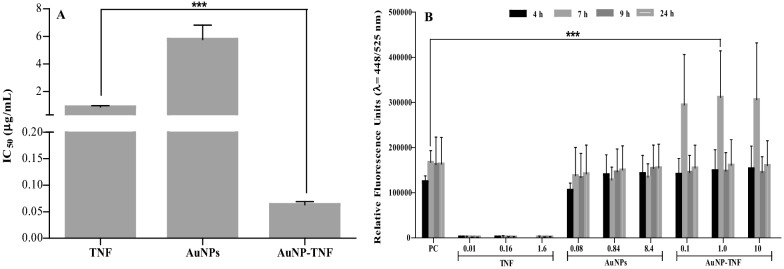
**A** Inhibitory effect of the free TNF, AuNPs, and AuNP-TNF on HIV-1 Reverse Transcriptase (RT-ase) activity. Data are expressed as mean±SD of IC_50_ (µg/mL). **B** Inhibitory effect of the free TNF, AuNPs, and AuNP-TNF on HIV-1 protease activity. Data are expressed as mean ± SD RFUs captured from GFP release in different groups, concentrations (µg/mL), and time points. Data is represented as three independent experiments. Statistical significance between the treated group of AuNP-TNF as compared to the free drug (TNF) in anti-HIV1 RT-as assay and with the anti-HIV1 protease enzyme control group (PC) in the is presented by an asterisk (***denotes p ≤ 0.0001)

The inhibitory impact of free TNF, AuNPs, and AuNP-TNF on HIV-1 protease was examined using His tagged-HIV protease. Figure 7B shows the mean ± SD of Relative Fluorescence Units (RFU) captured from Green Fluorescent Protein (GFP) release during 4–24 h after adding different concentrations (µg/mL) of the compounds to the enzyme. Compared to the PC (+ ve control), the three tested concentrations of AuNPs, and AuNP-TNF formulations suppressed HIV-1 protease activity. However, after 7 h of incubation with the enzyme, a higher level of released GFP was detected for AuNP-TNF, indicating that the AuNP-TNF had attained its optimal inhibitory effect on the enzyme. This experiment confirmed the multifunctional behavior of AuNPs in HIV-1 protease inhibition which alternatively attributed to suppressing the viral infection. (Fig. 7A).

##### Assessment of cellular uptake and internalization by Confocal Laser Scanning Microscopy (CLSM) and flow cytometry

**Fig. 8:**
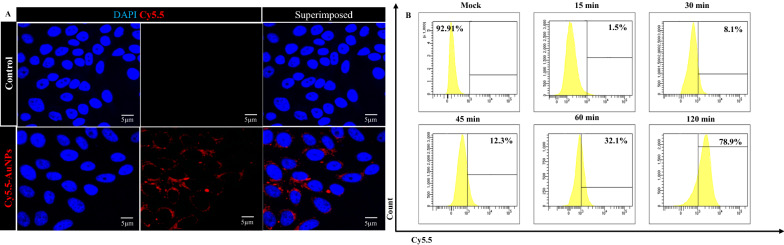
**A** Confocal images of cellular uptake of Cy5.5-AuNPs in TZM-bl cell line, **B** Flow cytometry analysis for assessing the time-dependent internalization of Cy5.5-AuNPs in the TZM-bl cell line

Confocal microscopy was used to examine the internalization capacity of Cyanine5.5 NHS ester tagged AuNPs (Cy5.5-AuNPs) by the TZM-bl cells. The cells had a distinctive red puncta hue, indicating an elevated accumulation of Cy5.5-AuNPs within the treated cells (Fig. 8A); in contrast, no such aggregation was seen in the mock cells. This finding adds to the evidence that TZM-bl cells can assimilate Cy5.5-AuNPs. The ingestion of Cy5.5-AuNPs by TZM-bl cells is shown in Fig. 8B. After 120 min of incubation with Cy5.5-AuNPs, the cells showed 78.9% absorption. The AuNPs were successfully ingested by the cells, as validated by these findings.

### In vivo* and *ex vivo* biodistribution*

**Fig. 9:**
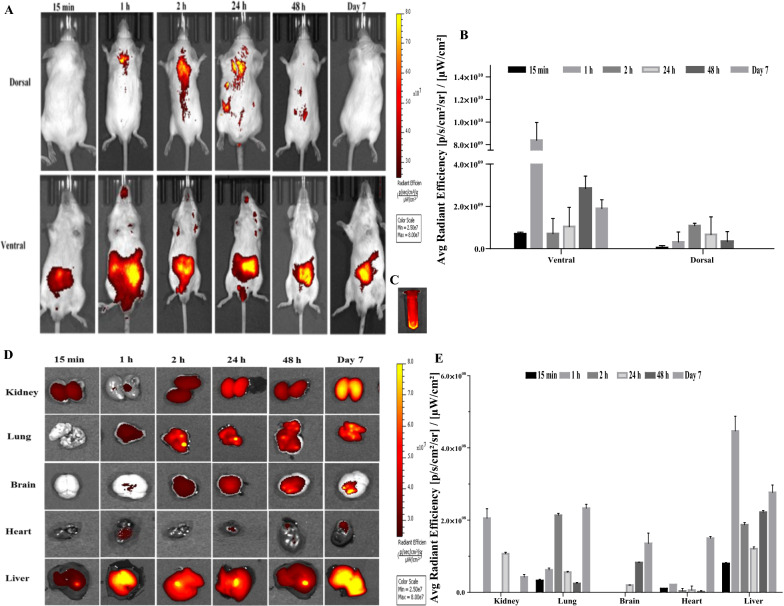
**A** Real-time whole-body images of the healthy male BALB/c mice, corresponding semiquantitative data of NIR fluorescence signal at prescheduled time point post-injection of Cy5.5-AuNPs, **B** Average radiant efficiency [p/s/cm^2^/sr]/[μW/cm^2^] of IVIS^®^ imaged mice. Data were obtained from the Region of Interest (ROI) of the fluorescent area in each mouse, **C** NIR fluorescence image of the Cy5.5-AuNPs stock in a tube, **D** displays images of dissected organs of healthy BALB/c mice post-injection of Cy5.5-AuNPs, **E** Average Radiant Efficiency [p/s/cm^2^/sr]/[µW/cm.^2^] of Cy5.5-AuNPs from isolated organs at different time point (Mean ± SD, n = 3)

For in vivo biodistribution analyses, the behavior of Cy5.5-AuNPs was evaluated in healthy male BALB/c mice by IVIS^®^ in vivo imaging system and Near-infrared (NIR) fluorescence images of major ex vivo organs (Kidney, Lung, Brain, Heart & Liver) were obtained at different time points (0.25 h, 1 h, 2 h, 24 h, 48 h and 7 days). All injected mice survived, and there were no visible signs of aberrant behavior or illness. At the injection location, there was no erythema or edema, indicating that there was no irreversible impairment to the endothelial cell structure or vessel wall due to the injection. Figure 9A depicts the NIR fluorescence images of the BALB/c mice at predetermined intervals following Cy5.5-AuNPs injection. As evident in Fig. 9A, the accumulation of the Cy5.5-AuNPs was more in the abdominal region 1 h after its administration. This observation was confirmed later on by ex vivo results. The NIR fluorescence signals were more intense in the liver and lungs. At 1 h, the rapid distribution of Cy5.5-AuNPs into the lungs and liver was observed. Notably, NIR fluorescence intensity was higher in the ventral than in the dorsal position.

Conversely, the accumulation of the targeted Cy5.5-AuNPs was more in the lung than in the kidney. In addition, the NIR fluorescence intensity increased in the brain over time. Targeted Cy5.5-AuNPs, on the other hand, tended to be more prevalent in the liver, which has higher metabolic activity.

3D Fluorescent Imaging Tomography (FLIT) was recorded 7 days post-administration that showed fluorescently tagged AuNPs were predominantly retained in the abdominal region (Additional file 2), which is an actual ex vivo observation of the trans-illumination of Cy5.5-AuNPs mainly in the Liver after 7 days in ventral position. To investigate Cy5.5-AuNPs accumulations in various organs ex vivo, the Average Radiant Efficiency [p/s/cm^2^/sr] / [µW/cm^2^] was determined (Fig. 9D). The obtained data were normalized by subtracting the values acquired by the background signal. As plotted in Fig. 9E, the accumulation of Cy5.5-AuNPs diminished in the kidney during the study period.

### Histopathology analysis

**Fig. 10:**
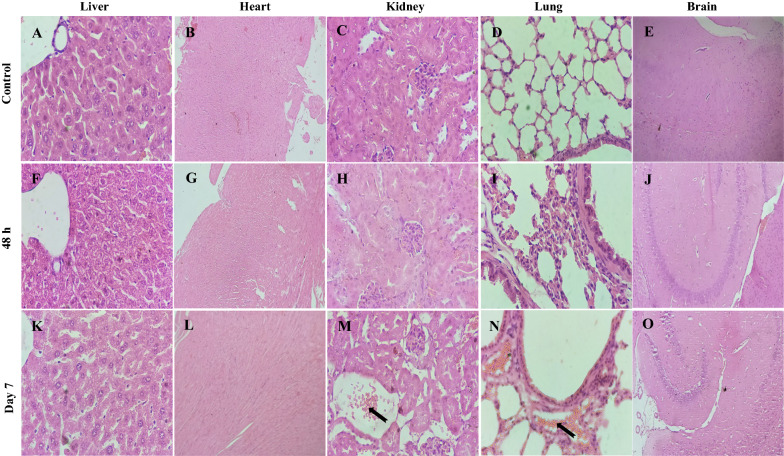
Light photomicrograph of histopathology of the major organs collected from the healthy BALB/c mice treated with 5 mg/kg of AuNPs at two-time points. (Magnification 40X for all images, except heart images taken at 10X magnification) Light micrographs of the organ sections from the different treatment groups. The letters on the images represent the treatment groups. **A**, **B**, **C**, **D**, and **E** No abnormality was detected in the control group. Control kidney section showing normal renal cortex and glomerular tufts, relatively healthy glomerulus with abundant capsular space. **F**, **G**, **H**, **I**, and **J** No abnormality was detected 48 h post-injection. **K**, **L** No abnormality detected, **M** Minimal focal peri-glomerular edema (arrow) observed in the kidney. **N** Minimal congestion of blood vessels (arrow) detected in the lung. **O** No abnormality was detected

Histopathology was carried out to examine the long-term in vivo biodistribution effects. This investigation aimed to see if the AuNPs had caused any tissue damage or inflammation in the major organs collected. Gross inspection of the retrieved organs and tissues revealed no abnormalities. The results (Fig. 10) suggested that treatment of nanoparticles did not show any abnormal histopathological changes in the liver, heart, and brain tissues of the mice post 48 h and 7 days of the injection of 5 mg/kg of AuNPs. However, microscopic examination of kidneys and lung exhibited that treatment of 5 mg/kg of AuNPs after 7 days caused minimal focal peri-glomerular edema (Fig. 10 N, arrow) and minimal congestion of blood vessels (Fig. 10O, arrow). Furthermore, despite observing the NIR fluorescence intensity in the heart and liver, the treatment could not cause any pathological changes compared to the untreated control group for 7 days suggesting that the AuNPs cargo was safe to be administered over the required schedule.

## Discussion

The present work aimed to develop a delivery agent for a hydrophilic anti-HIV1 drug, TNF. Due to their unique physical properties, we selected AuNPs as the delivery agent based on extensive published literature. Previous studies reported that AuNPs blocked viral entry/fusion and integrase activity [24, 25]. However, we stumbled upon some fascinating results. The ability of AuNPs to impede the reverse transcription process of the HIV replication cycle and the hitherto unknown protease inhibitor activity, which could stall virion maturation, are the true highlights of this work (see Graphical Abstract).

The citrate reduction method (employing sodium citrate to convert chloroauric acid to metallic gold) was used to synthesize AuNPs spherical AuNPs with narrow size distribution [54]. The AuNP-TNF were synthesized using the EDC/NHS cross-linking protocol for higher coupling efficiency and more stable amine-reactive intermediates. The conjugate yield via the EDC/NHS method was approximately 16%. The synthesized AuNPs and AuNP-TNF were characterized using DLS equipment as well as SEM and TEM imaging. An increase in the hydrodynamic size, as measured by DLS of AuNPs and AuNP-TNF from 26.5 nm to 37.6 nm, confirmed the successful conjugation of TNF on the surfaces of AuNPs. The ζ potential values (i.e., - 41.1 ± 5.94 mV and - 47 ± 5.77 mV for AuNPs and AuNP-TNF) suggested excellent stability of the synthesized nanoparticles. SEM and TEM analysis showed that AuNPs and AuNP-TNF were spherical with an average size of ~ 25 nm and ~ 31 nm, respectively, matching well with DLS data.

*UV-Vis* spectroscopy of the AuNPs showed a shift in the SPR peak from 524 to 526 nm and a change in the amplitude after TNF conjugation. Similar observations have been reported by many researchers [55]. Haiss et al*.* [56] reported a study for the determination of the size of NPs from the absorbance maximum in *UV-Vis* spectrum of AuNPs (A*spr*). The study demonstrated high accuracy between the calculated and measured sizes. The group further showed higher accuracy when using A*spr*/A450. They reported 1.8 and 1.96 A*spr*/A450 for 25 and 40 nm AuNPs, respectively which correlates well with present study wherein A*spr*/A450 for AuNPs and AuNP-TNF is 1.84 and 1.94, respectively [56]. The FT-IR (Fig. S3) and TGA (Additional file 1: Fig. S4) analyses also confirmed the successful conjugation of TNF with AuNPs [57, 58]. The synthesized AuNPs and AuNP-TNF were assessed for cell viability in different human cells. The primary screening of the preparations was carried out using a genetically-engineered HeLa cell clone (TZM-bl) for 48 h post-treatment. It is a standard in vitro protocol adopted by Montefiori et al*.* [59–62] to get an accurate and consistent assessment of cytotoxic concentration/s of the test agent/s used in the anti-HIV1 assays. In addition, we deployed PBMCs and MΦ cells to assess the long-term (typically, five days) impact of the preparations on the cell viability. These cells are the primary targets for the virus invasion and hence could mimic the in vivo scenario and help shed more light on the mechanisms underlying HIV infection [63–65]. The in vitro cell viability assay showed that CC_50_ values had a trend, i.e., AuNP-TNF>AuNPs>TNF in TZM-bl, PBMCs, and MΦ cells (Fig. 3). In a recent study, Surapaneni and co-workers showed that AuNPs of ~ 30 nm size exerted a cytotoxic effect on a MDA-MB-231 cells in a dose-dependent manner [66]. Our results (Additional file 1: Fig. S5) agree with the aforementioned data since our synthesized AuNPs induced a dose-dependent cytotoxicity in all cells. The most significant effect was observed at the highest tested concentration (600 µg/mL). When interpreting the results, it has to be taken into account that the associated cytotoxicity to AuNPs is significantly lower than the anti-HIV1 drug, TNF.

This work also employed a quantitative technique to measure apoptosis in the TZM-bl cell line after treatment with test compounds. We observed accelerated apoptosis in the AuNP- than AuNP-TNF treated cells. These findings are in line with the cell viability data. Several groups have reported that the AuNPs induce apoptosis due to generating the Reactive Oxygen Species (ROS) in an aqueous medium [67, 68]. However, the underlying mechanisms were not elucidated in these reports. On other hand, the in vivo fate of the AuNPs itself needs to be considered assessed the in vivo fate of AuNPs. The group reported that the AuNPs were degraded to ionic Au through action of ROS released by NADPH Oxidase (NOX) activity. The released Au ions are then sequestered by thiol containing molecule, metallothionein (MTs). A similar fate have been previously reported for the gold salts and small AuNPs (~ 4 nm). Thus, the study concluded that the in vivo fate of the gold salt and AuNPs is very similar. Based on these facts and the relatively small quantity of dose of AuNPs, we can conclude that there is high possibility of degradation of the AuNPs in vivo [69].

Hemocompatibility evaluation is critical because synthesized formulation interacts with blood, which is inevitable during in vivo administration. The hemolysis investigation suggested that the synthesized nano-ART exhibited lower hemolysis than the AuNPs.

In this study, AuNPs exhibited more pronounced cytotoxic, apoptotic, and hemolytic effects than their drug (TNF) counterparts. The observation is intriguing and needs in-depth analysis to unravel the underlying mechanisms. However, we conjecture that the characteristic findings are probably related to the size and shape of the AuNPs vis-à-vis environmental conditions. For example, AuNPs are known to show a 'protein-corona effect' in biological fluids. Also, there could be a slow release (leaching) of gold ions from AuNPs [70], exerting more potent effects such as enhanced cytotoxicity. Furthermore, the stress triggered due to uptake of gold ions in the RBCs could create a hypotonic environment and osmotic shock resulting in hemolysis [71–73]. On the other hand, when a drug masks AuNPs like TNF, the leaching of gold ions is diminished, and hence the resulting cytotoxic and hemolytic effects are less pronounced. However, we wish to emphasize that these conjectures need experimental confirmation. Although many studies on the genotoxicity of nanomaterials have been published, we could not find any literature focusing on an in-depth evaluation of AuNPs using primary cells like PBMCs. The PBMCs (i.e., lymphocytes and monocytes) are an essential element in the bloodstream. They circulate throughout the body and directly interact with many tissues/organs. In addition, these cells represent a host defensive mechanism that, when activated, can emit a spectrum of inflammatory mediators. Therefore, it is essential to mimic the interaction of nanomaterials with these cells [74]. However, simulating such interactions for AuNPs is challenging because gold is chemically inert, and hence theoretically, AuNPs would remain intact within the cellular structures for indefinite periods [75].

In this study, we adopted the alkaline comet assay to examine the potential induction of DNA damage caused by the interaction of PBMCs with nanomaterials. The fluorescent microscopy images (Fig. 4A) of EtBr-stained solo PBMC cells treated with test compounds and controls implied that comet tail length is a function of their concentrations and incubation time. Subsequently, the percent of DNA damage was quantified for each cell group treated with compounds and compared with controls. The findings from this assay suggest that AuNPs predominantly induced alkali-labile sites of the DNA and caused DNA damage. However, AuNP-TNF formulation showed marginally higher DNA damage than free AuNPs. Li et al*.* [76] observed that AuNPs exert genotoxic effects on the cells due to oxidative damage [76]. Therefore, the marginally higher DNA damage, as seen with AuNP-TNF in our study, could be logically attributed to TNF. Furthermore, TNF is known to exhibit potent DNA damage even at very low concentrations [77]. Thus, conjugation of AuNPs with TNF may outweigh the attenuation effect seen in our study (see Section genotoxicity) and manifests as marginally higher genotoxicity. The potential antiviral activity of the AuNPs upon conjugation with TNF was assessed in different cell types using an in vitro cell-associated anti-HIV1 assay. Initially, the primary screening was conducted in TZM-bl cells. Subsequently, to evaluate the prolonged interaction of the formulation in HIV-1 infected human cell lines, the antiviral activity was considered in MФ (human leukemia monocytic cell line, THP-1). These cells are not only hijacked by HIV but are recruited continuously to infect other areas essential in HIV immunopathogenesis. Therefore, the treatment for HIV infection must also target MФ in addition to lymphocytes. Hence, MΦ represents a robust and reproducible in vitro model to mimic the actual in vivo HIV infection scenario [78, 79]. One of the significant advantages of using THP-1 is that it differentiates into MФ in 2-3 days, whereas primary human monocytes do so in 5-10 days. Moreover, using MФ derived THP-1 cells was considered most appropriate as it reveals many biological characteristics of primary human monocytes/macrophages, such as modulation of protein phosphatase, Mg^2+/^Mn^2+^ dependent 1A (PPM1A) during pathogen invasion [80, 81]. In MФ, PPM1A functions as a checkpoint in the innate cellular immunological response to HIV infection [82]. In MФ, nano-ART cargo is an ideal drug transporter that might reach the target tissues/organs through a passive targeting strategy. Our anti-HIV1 assays against different HIV-1 strains showed that the AuNP-TNF improved the antiviral activity ~ 15 folds compared with the free TNF in all cells. More importantly, we found no significant difference in the HIV-1 inhibitory pattern against CXCR4 and CCR5 strains, underscoring the broad utility of nanosized formulation of TNF. Our data also indicated that the TNF inhibitory effect was not adversely affected upon conjugation to AuNPs, but enhanced the anti-HIV1 activity of TNF several folds. TNF as a NtRTI inhibits RT-ase, which converts the HIV-1 RNA into viral DNA and thereby halts the transcription step. Therefore, it is essential to factor in the activity of TNF while conjugating with AuNPs. Consequently, the HIV-1 reverse transcriptase in vitro assay verified that the TNF anti-HIV1 activity was not hindered while conjugating with AuNPs, which could explain the improved anti-HIV1 activity of this unique agent due to a synergistic effect of combining AuNPs and TNF.

In addition to the HIV-1 reverse transcriptase, the HIV-1 protease is a critical component of the HIV replication cycle at the post-entry stage. HIV-1 protease is a retroviral aspartyl protease (retropepsin), an enzyme involved with peptide bond hydrolysis in retroviruses [84]. In HIV, protease shepherds viral components to the host cell membrane, causing immature virions to emerge, which menacingly infect other cells. This enzyme has been extensively used as a potential therapeutic target since it recognizes a wide range of substrates. Thus, searching for novel protease inhibitors binding the catalytic site of the HIV protease to stall the emergence of immature virions is a promising strategy.

In the present study, we could observe an excellent anti-HIV1 activity of AuNPs and AuNP-TNF. Therefore, taking a cue from the above strategy, we thought it worthwhile to investigate if AuNPs and AuNP-TNF could have intrinsic protease inhibitor activity. Data presented in Fig. 7B clearly show that AuNPs and AuNP-TNF indeed possess excellent anti-protease activity, which strangely reduces after 7 h. These unusual but interesting results could probably be explained based on the unique size-shape-dependent properties of AuNPs and their ability to readily anchor to a broader range of ligands [85, 86]. Thus, AuNPs may interact with the protease, altering its conformation and preventing substrate attachment but are unable to sustain the effect beyond 7 h. However, we accept that the explanation is purely hypothetical at this stage and needs experimental confirmation. Nevertheless, we believe these results offer new hope for including AuNPs and AuNP-TNF in the armour of protease inhibitors in the crusade against HIV.

The range of the size of nanoparticles is critical for their cellular absorption, despite their nature or even cell charge and properties such as cell cycle phase [87] reported that the cellular ingestion of AuNPs was substantially size-dependent, with 50 nm nanoparticles demonstrating the maximum uptake by HeLa cells among a batch of AuNPs ranging in size from 10 to 100 nm [88]. Consequently, we have confirmed this ability. Flow cytometry analysis revealed that Cy5.5-tagged AuNPs could enter the cells with high efficacy post 2 h of treatment (78.9%). Furthermore, AuNPs were widely visualized in the cytoplasm using confocal microscopy, which confirmed the effective cytoplasmic delivery of AuNPs.

We evaluated the effect of in vivo* i.v.* administration of Cy5.5-AuNPs into healthy BALB/c mice by recording the results at different time points spread over seven days. The higher intensity of the NIR fluorescence signals in the liver and lungs observed at all the time points could be due to preferential uptake and retention of Cy5.5-AuNPs via the Mononuclear Phagocyte System (MPS). The MPS is mainly composed of resident macrophages. The macrophages are found in all the connective tissues, including the liver (i.e., Kupffer cells), lungs, and central nervous system (microglia) [89]. However, the loss of the persistent NIR fluorescence signals over time in the liver suggested that the rapid uptake of the Cy5.5-AuNPs was interrupted by macrophages in the Reticuloendothelial System (RES). It is noteworthy to mention the presence of Cy5.5-AuNPs in the abdominal region, which shows the clearance may depend mainly on the digestive system via hepatobiliary transport. A similar clearance mechanism was reported in a study by Zhang Y. et al*. *[89, 90]. Cy5.5-AuNPs in the kidney 7 days post-administration can be related to renal excretion trends of targeted AuNPs. Fluorescence intensity of Cy5.5-AuNPs in healthy BALB/c mice during the study period indicated the high permeability of AuNPs and ultimately confirmed biodistribution, and passive targeted delivery, suggesting the long-acting behavior of AuNPs. These results validated that the AuNPs could reach many tissues/organs, serving as a secure nest for HIV in the longer run. The results confirm our main objective to use this cargo as a long-acting agent to overcome deficient drug delivery to HIV reservoirs. Observation of the Cy5.5-AuNPs in the brain is attributable to their small size and high penetration efficacy. This study also revealed cargo permeability via the Blood-Brain Barrier (BBB), resulting in access to the Central Nervous System (CNS). HIV can penetrate the brain, creating a remote reservoir and leading to neurocognitive disorders [91]. This approach will be a potential application for the synthesized novel cargo to deliver TNF to the CNS to achieve viral suppression. Additionally, the histopathological data manifested that administration of Cy5.5-AuNPs to BALB/c mice did not induce tissue damage in 7 days.

Nevertheless, while implementing these nano Drug Delivery Systems (DDSs), attention must be given to factors such as the slower plasma clearance rate and their propensity to permeate through the biological barriers like BBB due to longer residence time [67].

## Conclusions

The results presented here prove that TNF-tethered AuNPs exhibit excellent anti-HIV1 activity, reduced toxicity to human cells, and an elevated therapeutic index. In addition, the preparation also showed potent anti-HIV1 protease activity, which is one of the primary targets in contemporary research on HIV therapeutics. These unique, hitherto unknown traits of AuNPs and TNF hold tremendous promise in facilitating simultaneous delivery of the therapeutics to the site of action and unreachable reservoirs of HIV to achieve virus eradication. The multifunctional activity of AuNP-TNF needs in-depth investigation to elucidate the underlying molecular mechanisms. Moreover, an extensive in vivo study using relevant HIV models to assess long-term biodistribution and effects of repeated dosage is warranted to take this novel preparation to the next stage of clinical trials. Besides, *in silico* modelling of the drug-target interaction might be fascinating. Finally, we believe that the work opens up new vistas for developing a variety of nanotechnology-based DDS, the new age silver bullets, to combat existing and emerging infectious diseases.

## Material and methods

### Materials

Tenofovir (≥ 98% HPLC) was procured from Clearsynth, Bangalore, India. Dulbecco's Modified Eagle Medium (DMEM), Roswell Park Memorial Institute (RPMI) 1640 medium, Penicillin-Streptomycin (Pen-Strep), Fetal Bovine Serum (FBS), Phosphate Buffered Saline (PBS) and HEPES buffer from Gibco, Invitrogen, New York, USA. Cyanine5.5 NHS ester, the amine-reactive far-red emitting fluorescent dye, was purchased from Abcam, Waltham, USA. Dimethyl sulfoxide (DMSO), 3-(4,5dimethyl thiazole-2-yl)-2, 5-diphenyl tetrazolium bromide (MTT), and Cysteamine hydrochloride ≥ 98% (titration) from Sigma Aldrich, Burlington, USA. The other chemicals were reagent/HLPC grade and were employed with no additional refinement.

### Gold nanoparticles synthesis

The citrate reduction process was used to synthesize AuNPs [92]. Before synthesis, all the glassware was rinsed with aqua regia and washed thoroughly with distilled water. Briefly, a 20 mL aqueous solution of 0.1 mg/mL Tetrachloroauric acid (HAuCl_4;_ 99% pure, Sigma-Aldrich, Burlington, USA) was used as a metallic precursor and allowed to heat to the point of boiling. Then 2 mL of the reducing agent, 1% sodium citrate solution, was added instantly and stirred for 10 min. Infographic, Fig. 1A, illustrates the reaction scheme for preparing AuNPs.

### Characterization of AuNPs and AuNP-TNF nanoconjugate

#### Particle size and surface charge analysis

Using DLS, the size and ζ potential were estimated. The hydrodynamic size and the surface charge of AuNPs and AuNP-TNF were measured by DLS and ζ potential, respectively. (Zeta sizer nano instrument, Malvern, Cambridge, UK), The nanoformulation was dispersed in water at a medium at 25 ℃. Clear disposable ζ cells were used for measurements were taken in triplicates, and mean particle size reported.

#### FE-SEM analysis

The size and morphology of the synthesized AuNPs were evaluated using FE-SEM. The FE-SEM images were captured at a 20 kV accelerating voltage to investigate the surface morphology of the synthesized AuNPs. (Nova NanoSEM^™^ 450; FEI, Hillsboro, USA). Briefly, a small amount of AuNPs was deposited over an aluminum substrate and pasted over double-sided carbon tape. Subsequently, the sputtered coated samples were analyzed. The EDS was carried out along with FE-SEM for qualitative and semiquantitative analysis of elements present in the materials under study (Bruker; XFlash^®^ 6I30, Karlsruhe, Germany).

#### TEM analysis

TEM is extensively used to image the size of nanoparticles, disclose phase/crystallographic orientation information via a diffraction pattern, and determine chemical composition via the energy spectrum. In this study, the size and morphology of the synthesized AuNPs were also evaluated using TEM. A drop of AuNPs was deposited on carbon film-covered copper mesh TEM grids, air-dried for 60 min, and then imaged. (Tecnai G2; U-twin, Hillsboro, USA).

#### Drug conjugation to AuNPs

Amine coupling through reactive esters is the most frequent strategy for connecting ligands covalently to a drug's hydrophilic solid surface, such as TNF. In this study, TNF was conjugated to AuNPs surface by using EDC and NHS coupling. Briefly, 2 mg AuNPs were activated by adding 6.96 µmol of EDC and NHS in an aqueous environment. The reaction mixture was stirred at ambient temperature for 2 h in the dark. (Fig. 1A).

Subsequently, 6.96 µmole of TNF solubilized in water was introduced to the reaction mixture and, for 24 h, stirred in the dark. (Fig. 1B) The conjugate was then pelleted by centrifugation at 12,400 g for 20 min and washed thrice with water to eliminate any unreacted reagents and by-products. The supernatant drug concentration was calculated by measuring absorbance at 259 nm [31]. The following Eq. (1) was used to compute the conjugation capacity:-

1 $$Conjugation\,capacity (\%) = (Total\,drug - drug\,in\,supernatant)/(total\,drug)\times 100$$

DLS was used to determine the size and surface charge of the AuNP-TNF. Using a *UV-Vis* spectrophotometer, the spectrum was recorded in the 400-700 nm range. Additionally, as described below, FT-IR was carried out in the 400-4000 cm^−1^ range.

#### *UV–-Vis* spectroscopy

*UV-Vis* absorption spectra of AuNPs and AuNPs conjugated TNF were recorded to determine the successful conjugation of TNF on the AuNPs surface. The *UV-Vis* analysis was performed using a *UV-VIS* spectrophotometer, Shimadzu 1800, Kyoto, Japan, in double beam standard quartz cuvettes with the spectral range of 400-700 nm wavelength at Room Temperature (RT); the *UV-Vis* absorption spectra were obtained [93]. Briefly, 5 μL of AuNPs or AuNP-TFV were diluted with Millipore water and analyzed by the spectrophotometer.

#### TGA analysis

TGA was used to evaluate bare AuNPs and TNF-conjugated AuNPs (PerkinElmer-STA 6000 Simultaneous Thermal Analyzer, Waltham, USA). Under flowing nitrogen, individual compounds were heated to 800 ℃ at 55 ℃/min while retained on an aluminum pan. In the presence of air, under the same heating rate from 800 ℃ to 1000 ℃, the compounds were then decomposed.

#### FT-IR spectroscopy

Additionally, AuNPs were analyzed with FT-IR (IRAffinity^−1^, Shimadzu, Columbia, USA). The AuNPs were dispersed in KBr pellets, and the samples were examined in transmission mode in the spectral band 400-4000 cm^−1^. The scanning speed was 20 mm/sec at RT, and the spectral resolution was 4 cm^−1^.

#### Synthesis of Cy5.5-tagged AuNPs

The synthesis was carried out as per the method reported in Wai and New (2020) [94]. Briefly, to 20 mL of 0.1 mg/mL HAuCl_4_ solution, 200 µL of 213 mM cysteamine hydrochloride was poured. Under dark conditions at RT, the mixture was vigorously stirred. Next, the solution was agitated for 10 min after 5 µL of freshly prepared cold 10 mM NaBH_4_ was added and then mildly stirred for an extra 30 min. The solution was stored overnight in the dark. Subsequently, Cy5.5 NHS ester was added to the AuNPs, swirled for 4 h in the dark, and then washed with distilled water. The Cy5.5-tagged AuNPs were used for internalization, uptake, and in vivo studies.

### In vitro* experiments*

#### Cell cultures

The genetically altered HeLa cell line, TZM-bl (JC53-bl), HEK 293 T, and Human Monocytic THP-1 cell lines were acquired from the National Institutes of Health (NIH), AIDS Research and Reference Reagent Program (ARRRP), Bethesda, USA. For primary screening of the synthesized nano-ART, the TZM-bl reporter cell line was employed. These cells express CD4, CXCR4, and CCR5 receptors, which are essential for HIV-infected cells. Additionally, Escherichia coli β-galactosidase enzyme and Tat-responsive reporter genes for firefly Luciferase (Luc) are inserted in these cells. The HIV-1 Long Terminal Repeat (LTR) promoter regulates the expression of these genes. This cell line was maintained in DMEM enriched with 25 mM HEPES buffer solution, penicillin (100 U/mL), streptomycin (100 mg/mL), and 10% FBS. Cell cultures were retained at 37 ℃ in a humidified atmosphere of 5% CO_2_. Adherent HEK 293 T cells were employed to propagate the infectious virus particles. HEK 293 T cells were also maintained under identical conditions as the TZM-bl cells. In addition, the THP-1 cells were also employed as an in vitro target cell system to simulate the efficiency of synthesized nano-ART during HIV infection. The cells were maintained in a complete RPMI 1640 growth medium fortified with 25 mM HEPES solution, 100 U/mL penicillin G, 100 mg/mL streptomycin, and 10% FBS. Further, naïve THP-1 cells were differentiated into MΦ by re-suspension in the complete growth medium, supplemented with 200 ng/mL (324 nM) phorbol 12-myristate 13-acetate (PMA; Sigma-Aldrich, Burlington, USA) for 2 or 3 days followed by 1 day of rest in PMA-free growth medium. Disseminated morphology, cell adhesion, increased granularity, and abnormal nucleus shape, which are hallmarks of THP-1 derived macrophages (MΦ), were promoted after the treatment, as observed by optical microscopy. Ficoll Histopaque^®^ (1077; Sigma-Aldrich, Burlington, USA) was used to separate PBMCs from the whole blood of a healthy donor using a density gradient centrifugation technique. PBMCs were activated with 5 µg/mL Phytohaemagglutin (PHA-p; Sigma-Aldrich, Burlington, USA) and maintained in RPMI 1640 medium enriched with 10 U/mL Interleukin-2 (IL-2; Roche, Branchburg, USA), and 10% FBS.

#### Propagation of viruses

##### HIV-1 primary isolates

In the present study, the anti-HIV1 activity against HIV1_VB28_ (CCR5 tropic, Subtype C isolate, Virus repository, ICMR- NARI, Pune, India) and HIV1_UG070_ (CXCR4 tropic, Subtype D [Uganda], NIH ARRRP, Bethesda, USA) was assessed. The activated PBMC cells were infected with the viral strains to develop virus stocks. The ELISA was conducted to monitor the viral growth using the HIV-1 p24 antigen detection kit (Advanced Bioscience Laboratories; Inc, Rockville, USA). Virus cell-free culture supernatants were harvested, centrifuged, filtered, and preserved at -80 ℃ in aliquots. The TCID_50_ (50% tissue culture infective dose) of each virus stock was calculated after titration.

##### The pseudotyped

The HIV-1 NL4-3 molecular clone (pNL4-3) encoding full-length HIV-1 was received from the NIH-ARRRP, Bethesda, USA. TOP10 competent cells were used to propagate the plasmid. (*E. coli* DH5 alpha; Invitrogen, New York, USA). Briefly, TOP10 competent cells were grown overnight on an incubator shaker at 37 ℃ in Luria Bertani broth enriched with ampicillin (100 µg/mL). Using a Qiaquick^™^ spin miniprep kit (Qiagen, Germantown, USA), the plasmid was extracted and quantified on a Nanodrop UV spectrophotometer. The medium was replenished with a fresh medium prior to transfection. Then, as per the manufacturer's protocol, 1.5 g of plasmid DNA was transfected into HEK 293 T cells (4 × 10^5^ /well) in a 6 well plate (Techno Plastic Products, Trasadingen, Switzerland) using X-tremeGene HP transfection reagent (Roche, Basal, Switzerland). The culture supernatant was collected, centrifuged, filtered (PALL^®^ syringe filter [0.22 µm]; Pall Corporation, New York, USA), and retained at -80 ℃ until needed. The Multiplicity of Infection (MOI) of the virus stock was determined, after which it was titrated in the MΦ.

### Cell proliferation assay

To measure cell viability, the tetrazolium-based MTT assay was executed. In TZM-bl, PBMCs, and MΦ, the CC_50_ values of free Tenofovir, Gold nanoparticles, and Gold nanoparticles conjugated with Tenofovir were determined. The TZM-bl cells (0.01e^6^ /well) were plated into flat-bottom 96 well microplates and stored at 37 ℃ in a humidified atmosphere of 5% CO_2_ overnight.

The naïve THP-1 cells were differentiated into MΦ in flat-bottom 96 well microplates as per the procedure described above. Subsequently, the medium was replenished with a serum-free medium. Following that, two-fold serial dilutions of free TNF, AuNPs, and AuNP-TNF were prepared and transferred into wells. The TZM-bl and MΦ cells plates were incubated for 48 h and 5 days at 37 ℃ in a humidified atmosphere of 5% CO_2_, respectively. The 0.2e^6^ /well of stimulated PBMCs (previously described) were seeded in U-bottom 96 well microplates and treated with various concentrations of compounds. The plates were incubated at 37 ℃ in a humidified atmosphere of 5% CO_2_ for 5 days. MTT solution (20 μL of 5 mg/mL strength) was added to each well after incubation. The plates were maintained for 4 h at 37 ℃ in similar conditions. Finally, the formed formazan crystals were dissolved with DMSO. At 550/630 nm, the Optical Density (OD) values were measured, and the results were represented in terms of CC_50_.

### Genotoxicity

Single-Strand DNA Breaks (SSBs) and DNA Double-Strand Breaks (DSBs) along with Alkali Labile Sites (ALS) were detected using the alkaline comet assay. This assay is widely referred to as "Single-cell micro-gel electrophoresis." Here, stimulated PBMCs were incubated with TNF, AuNPs, and AuNP-TNF at 5 and 150 μg/mL concentrations for 3 and 24 h, respectively, at 37 ℃ in a humidified atmosphere of 5% CO_2_. The stimulated PBMCs without treatment was considered a negative control, and cells exposed to 40 µg/mL Cyclophosphamide were regarded as a positive control. The medium containing treatment compound was replenished, and 50 µL of the mincing solution was added to each sample (10% DMSO and 20 mM EDTA in Hank's Balanced Salt Solution (HBSS, devoid of Ca^2+^, Mg^2+^, and phenol red, pH 7.5). The comet assay was carried out in accordance with the previously reported procedure [95, 96]. In short, 20 µL of cell lysate (0.2e^6^ cells) were combined with 150 μL of 0.5% Low Melting Point Agarose (LPMA; Sigma-Aldrich, Burlington, USA). Then, onto chilled microscope slides pre-coated with 1.0% Normal Melting Agarose (NMA), 20 μL aliquots of each sample were dropped. After adding newly prepared 1% Triton X-100 lysis buffer (2.5 M NaCl, 100 mM EDTA, 10 mM Tris Base, pH 10.0), the cells were lysed on the ice at 4 ℃ for 1 h. They were then immersed in an alkaline buffer (0.3 M NaOH, 1 mM EDTA, pH > 13.0) for 20 min to unwind. Subsequently, for 20 min, the electrophoresis was carried out in the same buffer at a power supply of 25 V and a current of 300 mA. The slides were then neutralized for 2 × 5 min in 0.4 M Tris buffer and maintained for 5 min in Milli Q water. The slides were fixed by dipping them for 5 min in chilled methanol and then dehydrated overnight. Eventually, by immersing air-dried slides in 1X EtBr (GeNei^™^, Bengaluru, India) for 15 min, the DNA was stained. A fluorescent microscope (AxioCam MRmImager 2^®^; Carl Zeiss, Oberkochen, Germany) was used to obtain the single-cell image. The results were then scored using the Comet Imager software (version 2.2) and analyzed with FIJI software. (Image J; Version 2.0.0, NIH, Bethesda, USA).

Fifty cells per sample were scored (fifty comets on each duplicate gel). The findings were represented as the average of the following parameters: % DNA damage and Olive Tail Moment (OTM). The OTM was calculated using the Eq. (2):-

2 $$Olive\,Tail\,Moment= (tail\,mean-head\,mean) \times \%\,of\,DNA\,in\,the\,tail$$

For each compound and concentration, three different experiments were carried out.

### In vitro* hemolysis assay*

AuNP-TNF must be distributed systemically to be effective HIV therapeutic, which is accomplished chiefly by blood circulation throughout the body. As a result, the nanoparticles' blood compatibility assessment is an effort to examine the nanoparticles' toxicity profile for Intravenous (*i.v.*) administration. A colorimetric hemolysis assay was used to assess the quantity of liberated red-colored hemoglobin, representing the RBCs disintegration level. The analysis was carried out according to the previously published protocol [63, 97, 98]. Thus, the in vivo scenario may be mimicked with this experiment.

Under medical supervision, the blood collected from a healthy donor is stabilized with EDTA and processed promptly. First, using centrifugation (800 g, 5 min), the RBCs were separated. Isolated RBCs were washed five times, pelleted, and resuspended in PBS. Subsequently, to acquire a 4% RBC suspension, the RBCs were diluted in a 5% w/v glucose solution. Finally, RBCs suspended in PBS and Triton-X 100 (1% v/v) were deployed as negative and positive controls, respectively.

TNF, AuNPs, and AuNP-TNF were incubated with RBC suspensions at concentrations of 5, 10, and 20 μg/mL. For 2 h, the suspensions were kept at 37 ℃ in a humidified atmosphere of 5% CO_2_.

Using a microplate reader, the OD of released hemoglobin was measured at 540 nm. The proportion of hemolysis was determined using the Eq. (3):-

3 $$\% Hemolysis = \left[ {\left( {A{-}B} \right)/\left( {C - B} \right)} \right] \times 100$$

where; A represents test OD, B represents negative control OD, and C represents positive control OD. For each term of the equation, the background interference was subtracted from each OD value. Three independent replicates of the experiment were carried out.

### Annexin V‑FITC/Propidium Iodide (PI) apoptosis assay

TZM-bl cells (1 × 10^6^/well) were treated with TNF, AuNPs, and AuNP-TNF at 80, 420, and 500 µg/mL concentrations, respectively. According to the manufacturer's procedure (Annexin V-FITC/PI Apoptosis Assay kit; Invitrogen, New York, USA), staining of the treated cells was done for 10 min at RT in the dark with 5 µL PI for 5 min and 5 µL Annexin V-FITC. Cells without treatment served as a negative control, and cells treated with 10 µM Camtothectin (CPT), an inhibitor of DNA topoisomerase that induces Programmed Cell Death (PCD), served as a positive control. Using a FACSAria^™^ Fusion flow cytometer (Becton Dickenson, Franklin Lakes, USA), the amount of apoptosis induced by compounds in TZM-bl cells was quantified. In addition, apoptosis was evaluated using FlowJo software (version 10.0). The results were presented as the overall apoptosis rate (the early and late apoptotic cell population).

### Virucidal assay

The efficacy of free TNF, AuNPs, and AuNP-TNF to inhibit HIV-1 replication was assessed in different HIV-1 infected cells. The TZM-bl cells (0.01e^6^ /well) plated on the previous day were infected with the pre-titrated viral stocks of HIV1_VB28_ or HIV1_UG070._ The plates were incubated for 2 h. This was followed by adding two-fold serial dilutions of the sub-toxic concentrations of free TNF, AuNPs, and AuNP-TNF. The plates were then incubated at 37 ℃ in a humidified atmosphere of 5% CO_2_ for 48 h. The cells grown without virus (100% inhibition) were considered the negative control, whereas the cells inoculated with the virus without compounds (100% infection) were considered the positive control. Post incubation, the Britelite plus reagent (Perkin Elmer, Waltham, USA) was added, the Relative Luminescence Units (RLUs) were quantified by a Luminometer (Victor 3; Perkin Elmer, Waltham, USA), and the percent inhibition was measured by the LUC software (version 04.4). The results were analyzed in terms of IC_50_. The TIs ($TI= CC{50}/IC{50}$) were calculated and compared with the free TNF.

For confirmation of the anti-HIV activity, the activated PBMCs (0.02e^6^/well) were inoculated with HIV-1 primary isolates (R5, HIV1_VB28_, and X4, HIV1_UG070_), and the experiments were conducted using similar procedures and conditions as described above. The supernatant was collected after 5 days of incubation and analyzed for HIV-1 capsid p24 antigen. The findings were represented as IC_50_ and TI, and the percent inhibition was measured.

The anti-HIV1 efficacy of free TNF, AuNPs, and AuNP-TNF has also been assessed in MΦ cells. The cells (0.02e^6^/well) were infected with a pre-titrated pNL4-3 stock (MOI of 0.01) and incubated at 37 ℃ in a humidified atmosphere of 5% CO_2_ for 4 h. Each well's media was aspirated, and the cells were rinsed at least thrice with fresh serum-free RPMI 1640 medium. Then, different concentrations of free TNF, AuNPs, and AuNP-TNF were added to the cells. The supernatant from respective wells was collected on days 1, 3, and 5 post-infection. The collected supernatants were analyzed for the p24 Gag protein using an HIV-1 p24 antigen detection kit to determine the antiretroviral efficacy of compounds at each time point. Each experiment included untreated and untreated HIV-1 infected cells as negative and positive controls. Three independent replicates of the investigation were carried out, and the results were expressed as IC_50_ for the luminescent cell-based assay and HIV-p24 antigen levels (pg/mL).

### In vitro off-target activity evaluation to elucidate the mechanism of action

Enzymatic assays were carried out to understand the mode of action of free TNF, AuNPs, and AuNP-TNF in the HIV-1 triggered cells.

#### HIV-1 RT-ase assay

In accordance with the manufacturer's instructions, a colorimetric HIV-1 Reverse Transcriptase Assay (Roche, Mannheim, Germany) was employed to validate the activity. The potential of each compound to hinder the HIV-1 RT-ase enzyme was tested at various concentrations [99]. In a nutshell, for 60 min, the HIV-1 RT-ase and template nucleotide cocktail were incubated with different concentrations of compounds. Then, the mixtures were shifted to streptavidin-coated microtiter plates for an additional hour of incubation. As a result, the biotin and DIG-labelled template primer complex adhered to the streptavidin plates. The HRP enzyme conjugate was then added and incubated for an hour after rigorously rinsing to ensure that no unbound template remained. Absorbance at 405 nm and reference at 490 nm was measured after the substrate was added. The IC_50_ values were calculated. The IC_50_ values were calculated.

#### HIV-1 protease assay

The HIV Protease Activity Detection Kit (Sigma-Aldrich, Burlington, USA) was used to detect the inhibitory potential of AuNPs against HIV-1 protease. This kit is specific for the HIV Protease, a retroviral aspartyl protease critical for the HIV life-cycle. Briefly, a 1:1 master mix of the constrained substrate and Tris-EDTA (TE) buffer (pH 2.0) was prepared. Then, 20 µL of the master mix was pipetted to each microplate well. Later, 0.5 µL of His tagged HIV protease, recombinant from HIV-1 (Sigma-Aldrich, Burlington, USA) added to the positive control and experimental reaction wells. The microplate was incubated overnight at 37 ℃ in a humidified atmosphere of 5% CO_2_. Finally, 200 µL of the detector was added to each well, and the plate was incubated for 4-24 h at 37 ℃ in a humidified atmosphere of 5% CO_2_. At the excitation/ emission of 448/525 nm, the released GFP was measured. The blank fluorescence values were subtracted from the final fluorescence values of the sample(s) and the positive control. The data were obtained from three separate experiments, and the mean ± SD of RFU was determined.

### Investigation of cellular uptake and internalization

#### Confocal laser scanning microscopy (CLSM)

In 6-well plates, TZM-bl cells (0.1e^6^/well) were plated on Poly-L-Lysine (Sigma-Aldrich, Burlington, USA) pre-coated coverslips and were incubated overnight at 37 ℃ in a humidified atmosphere of 5% CO_2_. The medium was replenished to a serum-free medium with 0.2 mg/mL of Cy5.5-AuNPs. The medium was aspirated after 2 h of treatment, and the treated cells were rinsed three times with PBS. Subsequently, the cells were fixed in 4% Paraformaldehyde (PFA; Sigma-Aldrich, Burlington, USA) in PBS (pH 7.4). For 3 min, the cells were permeabilized with 0.1% Triton^™^ X-100 (Sigma-Aldrich, Burlington, USA) in PBS. The washed cells were then stained with 1 ng/mL DAPI (for nuclei; Sigma-Aldrich, Burlington, USA). After staining, the coverslips were rinsed, dried, and mounted on slides using ProLong^™^ Gold Antifade Mountant (Invitrogen, New York, USA). Cells were imaged with oil using CLSM (Olympus FLUOVIEW FV3000, Tokyo, Japan) at 60X magnification. The images were analyzed using cellSens Dimension with multichannel 5D software.

#### Flow cytometry analysis (FCM)

Cy5.5-AuNPs were deployed as a tracer, and the internalization of AuNPs in cells was measured by flow cytometry. TZM-bl cells (0.1e^6^ /well) were plated in 6-well plates and incubated for 24 h at 37 ℃ in a humidified atmosphere of 5% CO_2_ to form a confluent monolayer. The culture medium was swapped with serum-free DMEM the following day, and the cells were allowed to rest for 30 min. Next, to induce cellular internalization of the nanoparticles, 0.2 mg/mL of the Cy5.5-AuNPs were dispersed in serum-free DMEM and introduced to the cells. The plates were then incubated for 0, 15, 30, 45, 60, and 120 min at 37 ℃ in a humidified atmosphere of 5% CO_2_. Finally, the cells were rinsed three times with PBS to eliminate free Cy5.5-AuNPs before being detached using Trypsin-EDTA 0.05% (Gibco-Invitrogen, New York, USA). For acquisition, the collected cells were resuspended in 500 μL PBS in a FACS tube and analyzed using a FACSAria^™^ Fusion flow cytometer (Becton Dickinson, Franklin Lakes, USA) to quantify cellular uptake. The data analysis was conducted using FACSDiva^™^ software. Three independent replicates of the experiment were carried out.

### In vivo* and *ex vivo* biodistribution*

All studies were conducted following the guidelines of the Committee for the Purpose of Control and Supervision of Experiments on Animals (CPCSEA), Department of Animal Husbandry and Dairying (DAHD), Ministry of Fisheries, India. Ethical approval was obtained from the Institutional ethics committee (IEC) of the ICMR-NARI, Pune, India, and the Institutional Animal Ethics Committee (IAEC) of the Indian Institute of Science Education and Research (IISER), Pune, India. All mice utilized in this investigation were retained in standard dwelling conditions. They have been kept in Individually Ventilated Cages (IVC) with a 12 h light/dark cycle. The water and food were provided ad libitum.

In healthy mice, in vivo fluorescence imaging was employed to observe the real-time distribution of Cy5.5-AuNPs. This pre-clinical study is important to evaluate the efficacy of the synthesized AuNPs in reaching the tissues/organs. A single dose of 0.2 mL of Cy5.5 NHS ester-AuNPs (5 mg/kg, n = 3 for each timepoint) or PBS (control, n = 1 for each timepoint) was administrated intravenously to seven weeks old male BALB/c mice (≈ 25 g of weights). Using an in vivo imaging system (IVIS Spectrum; PerkinElmer, USA), the fluorescence distribution of whole-body (mice were under isoflurane) was captured at various time intervals post *i.v.* administration (0.25 h, 1 h, 2 h, 24 h, 48 h, and 7 days). The IVIS^®^ imaging system's scanning parameters were configured to emission/excitation at 673/707 nm, having a field of view of 13.5 cm and a fluency rate of 2 mW/cm^2^. Mice were sacrificed humanely at each time point following the scan. NIR fluorescence pictures of ex vivo organs were acquired after the main organs, the liver, heart, kidney, lung, and brain, were excised (673em/707ex filters). Using Living Image^®^ 4.72 software (64-Bit), the acquired pictures were processed, and Regions of Interest (ROI) were generated. After subtracting the background signal, the average radiant efficiency [p/s/cm^2^/sr]/ [W/cm^2^] was determined. The harvested organs were preserved in 10% neutral buffered formalin (Sigma-Aldrich, Burlington, USA) for histopathology analysis. FLIT was also recorded, which leverages the signal's geometry, depth, and intensity to create a 3D reconstruction of the mouse and provides anatomical localization of the fluorescence signal [100, 101].

### Histopathological staining and analysis

The organs of mice collected at each time point were fixed with 4% PFA, embedded in paraffin, sliced into thin sections, and stained with Hematoxylin/Eosin (H&E). Using a digital microscope, the slides were examined. For histological examination, each tissue (liver, heart, kidney, lung, and brain) was immersed in a 10% buffered neutral formaldehyde solution for 48 h. The tissues were dehydrated and embedded in paraffin once the chemical fixation was accomplished. A microtome was used to cut thin tissue sections with a thickness of 4-5 μm (Leica RM 2025; Wetzlar, Germany). Subsequently, the paraffin coat was dissolved in a water bath and closed on slides coated with Poly-L-Lysine. After being placed in an oven at 37 ℃, the sections were deparaffinized in xylol, and dehydrated by lowering the percentage of ethyl alcohol in the alcohol series. Finally, they were stained with H&E staining. The optical microscope (Olympus CH40; Tokyo, Japan) and a digital camera were used to inspect the slides.

### Statistical analyses

All experiments were performed in triplicate, and means with standard errors were computed. The statistical significance of differences in values between the study groups and controls was determined using one-way ANOVA. Differences with p ≤ 0.05 were regarded as statistically significant. GraphPad Prism 5 for Windows (GraphPad Software, USA) was used to conduct all statistical analyses.

## Supplementary Information

**Additional file 1: Fig. S1 **Recorded *UV-Vis* spectra of AuNPs (turquoise peak) and AuNP-TNF (green peak). **Fig. S2 **FE-SEM/EDS images of AuNPs; **(A-D)** EDS mapping images of Au powder and overlap elements.** (E)** EDX spectrum of AuNPs. **Fig. S3 **FT-IR spectra of AuNP-TNF, AuNPs, and free TNF. **Fig. S4 **TGA analysis of AuNP-TNF (black color) and AuNPs (blue color). **Fig. S5 **Effect of various concentrations (5-600 µg/mL) for TNF, AuNPs. And AuNP-TNF on cell viability using MTT assay in; **(A)** TZM-bl cell line, **(B)** PBMCs, and **(C) **MФ. The study showed the correlation between concentrations of gold nanoparticles and gold nanoparticles conjugated TNF on viability of different cells. Results are shown as mean±SD. **Fig. S6 **Hemolysis assay of free TNF, AuNPs and AuNP-TNF. Results are shown as mean±SD. **Fig. S7 (A)** Size and **(B)** Surface charge as measured by DLS of Cy5.5-AuNP. **Fig. S8 **Transmission Electron Microscopy [SAED image in inset] of AuNP-TNF.

**Additional file 2.** 3D Fluorescent Imaging Tomography (FLIT) record of the abdominal region of a BALB/c mouse, 7 days post-administration of fluorescently tagged AuNPs.
