# Supplementary material for: Tenofovir-tethered gold nanoparticles as a novel multifunctional long-acting anti-HIV therapy to overcome deficient drug delivery-: an in vivo proof of concept
Source: J Nanobiotechnology. 2023 Jan 19;21:19. doi: 10.1186/s12951-022-01750-w (PMC9850711; doi:10.1186/s12951-022-01750-w)
Supplement: Supplementary file 1 — Additional file 1: Fig. S1 Recorded UV-Vis spectra of AuNPs (turquoise peak) and AuNP-TNF (green peak). Fig. S2 FE-SEM/EDS images of AuNPs; (A-D) EDS mapping images of Au powder and overlap elements. (E) EDX spectrum of AuNPs. Fig. S3 FT-IR spectra of AuNP-TNF, AuNPs, and free TNF. Fig. S4 TGA analysis of AuNP-TNF (black color) and AuNPs (blue color). Fig. S5 Effect of various concentrations (5-600 µg/mL) for TNF, AuNPs. And AuNP-TNF on cell viability using MTT assay in; (A) TZM-bl cell line, (B) PBMCs, and (C) MФ. The study showed the correlation between concentrations of gold nanoparticles and gold nanoparticles conjugated TNF on viability of different cells. Results are shown as mean±SD. Fig. S6 Hemolysis assay of free TNF, AuNPs and AuNP-TNF. Results are shown as mean±SD. Fig. S7 (A) Size and (B) Surface charge as measured by DLS of Cy5.5-AuNP. Fig. S8 Transmission Electron Microscopy [SAED image in inset] of AuNP-TNF. [file 12951_2022_1750_MOESM1_ESM.docx]

**Additional file 1**

**Tenofovir-tethered gold nanoparticles as a novel multifunctional long-acting anti-HIV therapy to overcome deficient drug delivery­-: an *in vivo* proof of concept**

**Leila Fotooh Abadi^1^, Pramod Kumar^2^, Kishore Paknikar^2, 3*^, Virendra Gajbhiye^2,*^ and Smita Kulkarni^1*^**

^1^ Division of Virology, Indian Council of Medical Research-National AIDS Research Institute, Pune-411 026, India.

^2^ Nanobioscience Group, Agharkar Research Institute, Pune-411 004, India.

^3^ Department of Chemistry, Indian Institute of Technology, Powai, Mumbai-400 076, India.

***Corresponding authors Tel.: +91-20-27331207, Fax: +91-20-27121071**

**E-mail addresses:** [skulkarni@nariindia.org](mailto:skulkarni@nariindia.org), [smitak.2005@gmail.com](mailto:smitak.2005@gmail.com) (Dr. Smita Kulkarni), [virendragajbhiye@aripune.org](mailto:virendragajbhiye@aripune.org) (Dr. Virendra Gajbhiye), [kpaknikar@gmail.com](mailto:kpaknikar@gmail.com), [kpaknikar@iitb.ac.in](mailto:kpaknikar@iitb.ac.in) (Dr. Kishore Paknikar).

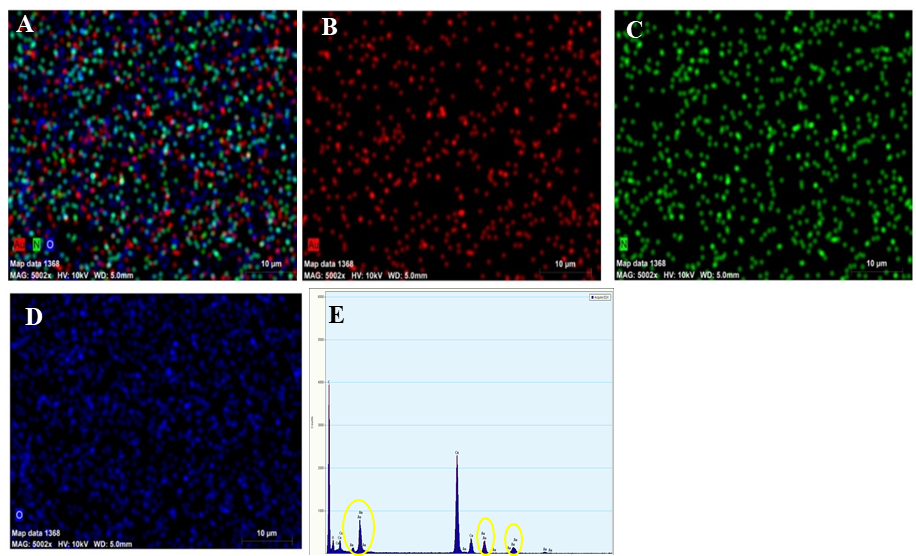
**Fig. S1** Recorded *UV-Vis* spectra of AuNPs (turquoise peak) and AuNP-TNF (green peak).

**Fig. S2** FE-SEM/EDS images of AuNPs; **(A-D)** EDS mapping images of Au powder and overlap elements. **(E)** EDX spectrum of AuNPs.

**Fig. S3** FT-IR spectra of AuNP-TNF, AuNPs, and free TNF.

**Fig. S4** TGA analysis of AuNP-TNF (black color) and AuNPs (blue color).

**Fig. S5** Effect of various concentrations (5-600 µg/mL) for TNF, AuNPs. And AuNP-TNF on cell viability using MTT assay in; **(A)** TZM-bl cell line, **(B)** PBMCs, and **(C)** MФ. The study showed the correlation between concentrations of gold nanoparticles and gold nanoparticles conjugated TNF on viability of different cells. Results are shown as mean±SD.

**Fig. S6** Hemolysis assay of free TNF, AuNPs and AuNP-TNF. Results are shown as mean±SD.


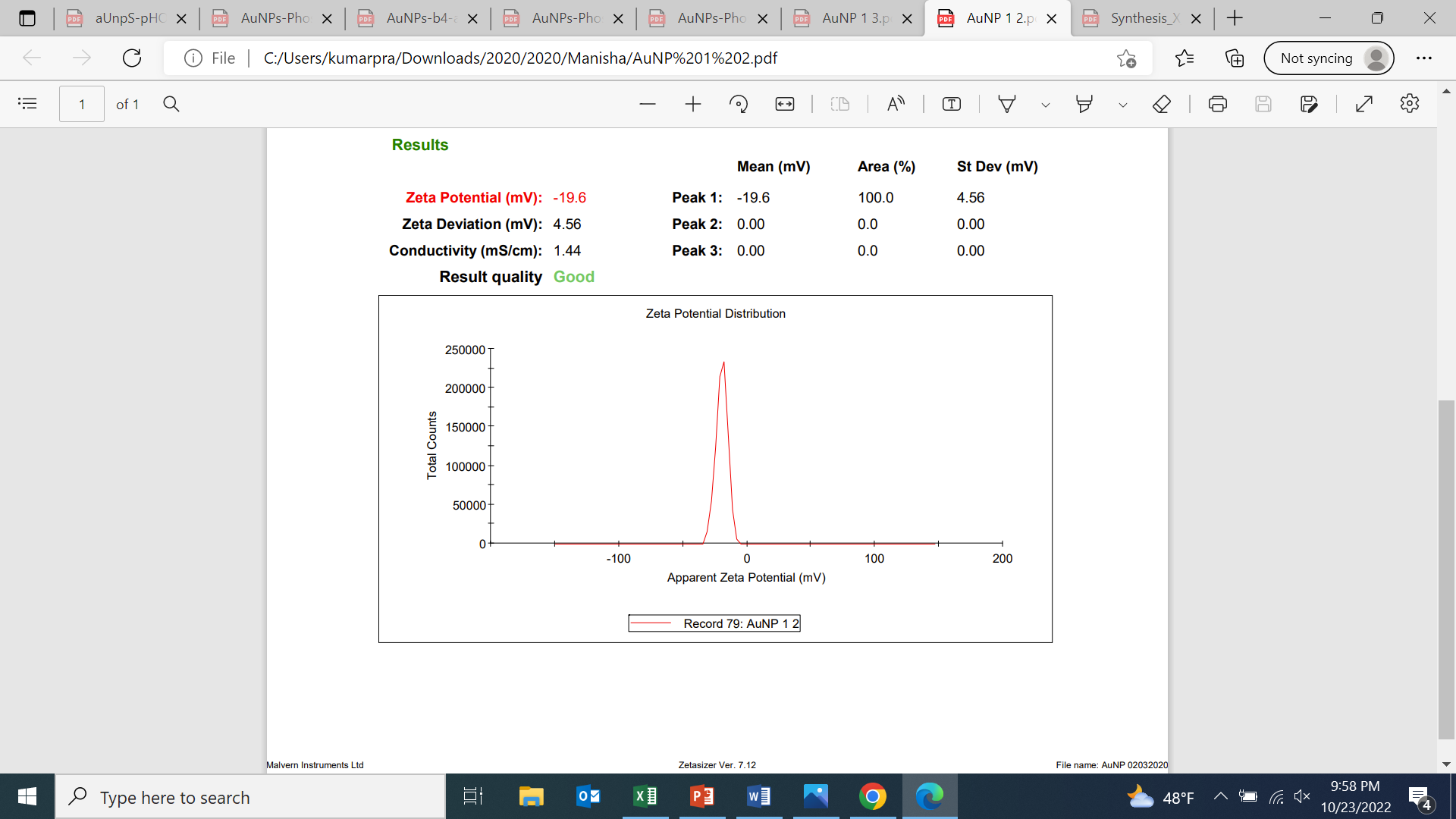


**-19.8±4.56 mV**


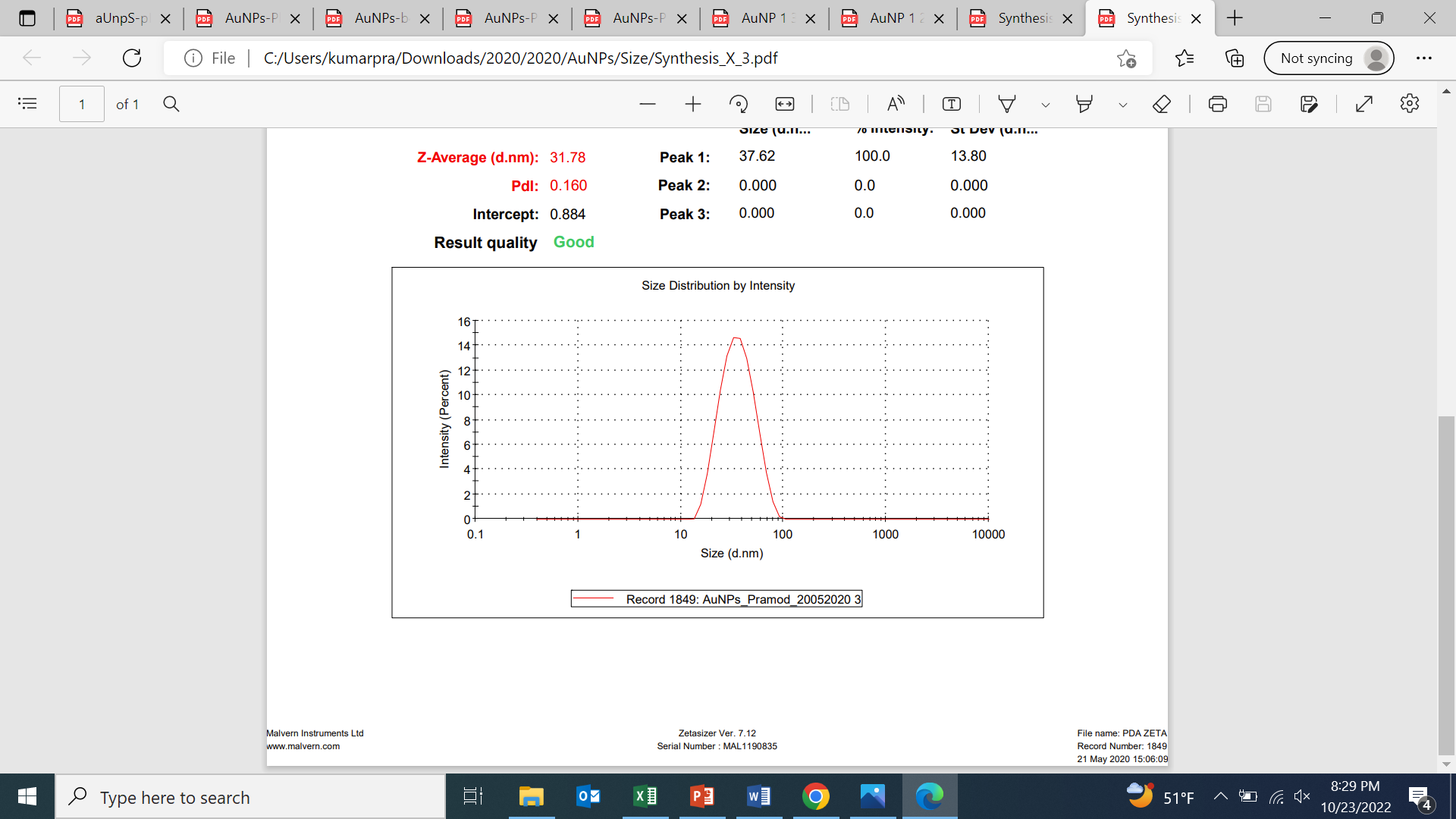


**31.78±4.8 nm**

**PDI- 0.160**

**A**

**B**

**Fig. S7 (A)** Size and **(B)** Surface charge as measured by DLS of Cy5.5-AuNP.


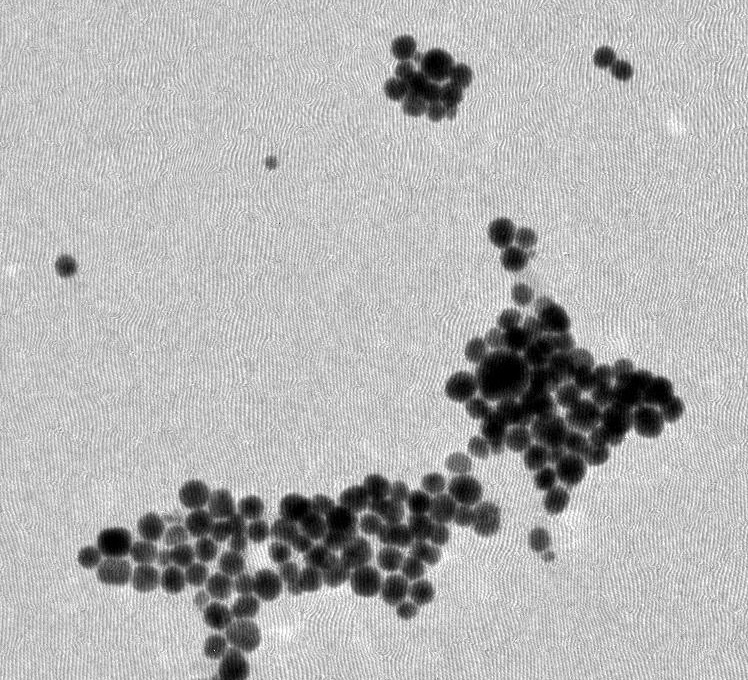

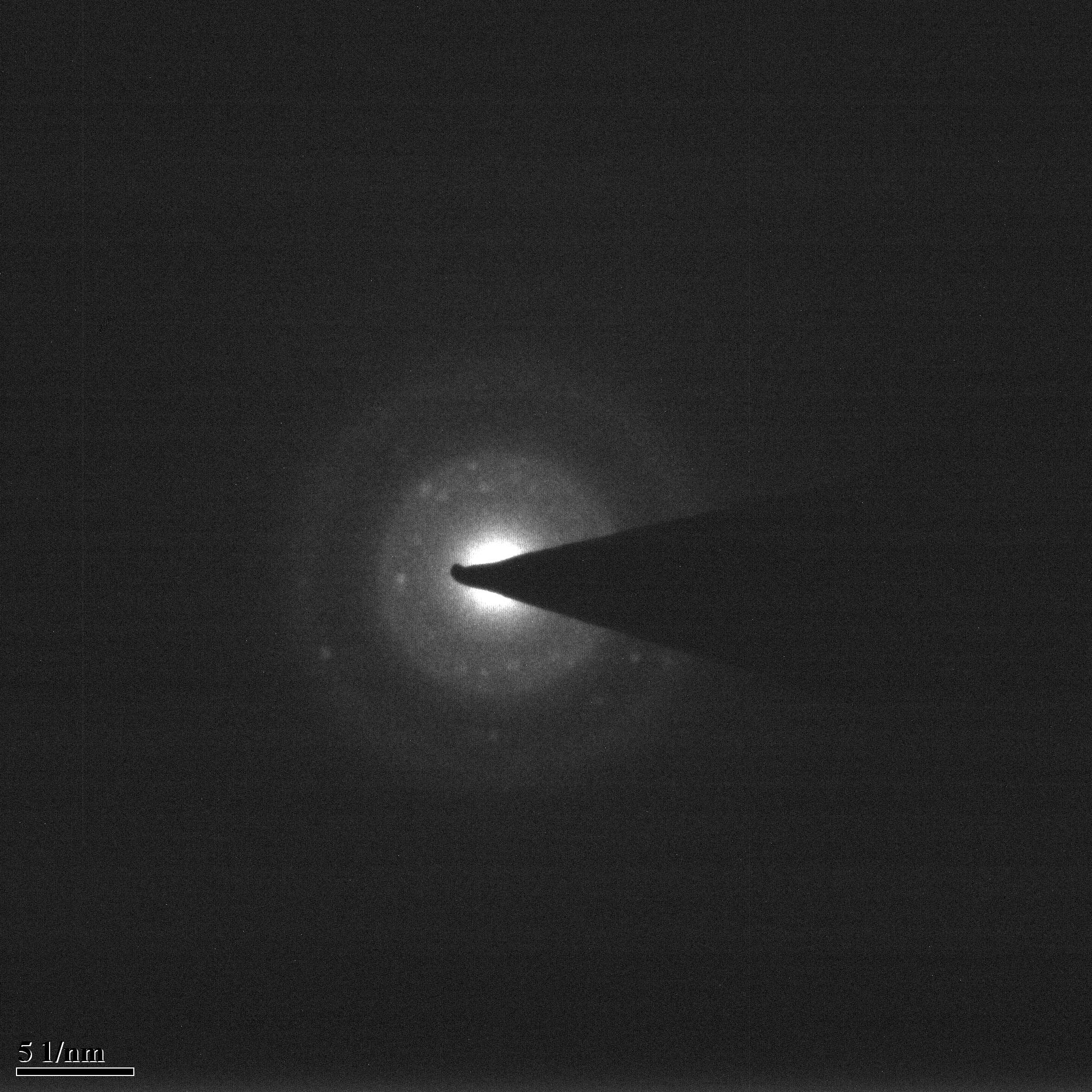


**Fig. S8** Transmission Electron Microscopy [SAED image in inset] of AuNP-TNF.
